# ERAS, a Member of the Ras Superfamily, Acts as an Oncoprotein in the Mammary Gland

**DOI:** 10.3390/cancers13215588

**Published:** 2021-11-08

**Authors:** Cristian Suarez-Cabrera, Isabel Ojeda-Perez, Raquel Sanchez-Baltasar, Angustias Page, Ana Bravo, Manuel Navarro, Angel Ramirez

**Affiliations:** 1 Translational Oncology Division, Centro de Investigaciones Energéticas, Medioambientales y Tecnológicas (CIEMAT), 28040 Madrid, Spain; cristian.suarez@ciemat.es (C.S.-C.); Isabel.Ojeda@ciemat.es (I.O.-P.); raquel.sanchez@ciemat.es (R.S.-B.); a.page@ciemat.es (A.P.); 2 Centro de Investigación Biomédica en Red de Cáncer (CIBERONC), 28040 Madrid, Spain; 3 Instituto de Investigación Sanitaria Hospital 12 de Octubre (imas12), 28041 Madrid, Spain; 4 Breast and Gynecological Cancer Group, Instituto de Investigación Sanitaria Hospital 12 de Octubre (imas12), 28041 Madrid, Spain; 5 Research Group on Pathology Phenotyping of Transgenic Mouse as Model of Human Diseases (GI-1845), Veterinary Pathology, Department of Anatomy, Animal Production and Veterinary Clinical Sciences, Veterinary Faculty of Lugo, University of Santiago de Compostela, 27002 Lugo, Spain; ana.bravo@usc.es

**Keywords:** ERAS, transgenic mice, Ras pathway, breast cancer, adenomyoepithelioma

## Abstract

**Simple Summary:**

The genes of the RAS family are among the group of genes most frequently mutated in human cancer. ERAS is a relatively unknown gene of this family. Although ERAS is overexpressed in some tumoral samples and in several cancer cell lines of human origin, it is not known if its expression drives tumor formation or if, alternatively, its expression is a secondary event in tumoral transformation. In this report, in order to clarify the role of ERAS in mammary tumorigenesis, we studied transgenic mice expressing ERAS in myoepithelial cells of mammary and other exocrine glands and in basal cells of stratified epithelia. These mice displayed an altered development and function of the mammary glands, and suffered high-frequency tumoral lesions in the mammary glands resembling a rare human breast tumor named malignant adenomyoepithelioma. Our results clearly demonstrate that ERAS is a true oncogene able to produce mammary tumors when inappropriately expressed.

**Abstract:**

*ERAS* is a relatively uncharacterized gene of the Ras superfamily. It is expressed in ES cells and in the first stages of embryonic development; later on, it is silenced in the majority of cell types and tissues. Although there are several reports showing ERAS expression in tumoral cell lines and human tumor samples, it is unknown if ERAS deregulated expression is enough to drive tumor development. In this report, we have generated transgenic mice expressing ERAS in myoepithelial basal cells of the mammary gland and in basal cells of stratified epithelia. In spite of the low level of ERAS expression, these transgenic mice showed phenotypic alterations resembling overgrowth syndromes caused by the activation of the AKT-PI3K pathway. In addition, their mammary glands present developmental and functional disabilities accompanied by morphological and biochemical alterations in the myoepithelial cells. These mice suffer from tumoral transformation in the mammary glands with high incidence. These mammary tumors resemble, both histologically and by the expression of differentiation markers, malignant adenomyoepitheliomas. In sum, our results highlight the importance of *ERAS* silencing in adult tissues and define a truly oncogenic role for *ERAS* in mammary gland cells when inappropriately expressed.

## 1. Introduction

Ras proteins, which belong to the large family of small GTPases, act as molecular switches in the signal transduction from extracellular signals that activate receptors with tyrosine-kinases activity (RTKs) to the nucleus. Ras GTPases achieve this by changing between the inactive GDP-bound and active GTP-bound states; the conversion between these states is regulated by GTPase-activating proteins (GAPs), such as NF1 and RASA1, and guanine nucleotide exchange factors (GEFs), such as SOS (reviewed in [1]). Ras proteins control a number of basic cellular functions whose alterations can lead to tumor development, as proliferation, differentiation, cell adhesion and apoptosis. Data contained in The Cancer Genome Atlas indicate that the Ras pathway is altered in more than 45% of human tumors [2]. Based on the databases of cancer mutations, it has been estimated that 19% of cancer patients harbor mutations in the three main RAS genes, i.e., *H-*, *N*- and *K-RAS* [3], *K-RAS* being the most frequently mutated gene in human cancer. In addition to these three main Ras members, there are around other 30 additional members in this family, whose role in cancer is less known [4].

ERAS (Embryonic Ras) is a Ras family gene member located both in humans and mice in the X-chromosome and, due to its particular sequence in critical regulatory residues (Ser instead of Gly in the conserved residue “Gly12” present in most of RAS genes), it is insensitive to GAPs and hence constitutively activated [5]. It is expressed in embryonic stem (ES) cells of mice, where it seems to have a role in promoting tumor-like properties [5] and in early embryonic development [6], being later silenced by epigenetic mechanisms, namely promoter methylation and histone acetylation [7,8]. Its expression pattern appears to be somewhat species-specific, as ERAS is expressed in primate non-tumoral ES cells [9] but not in human ES cells [10]. Its study in human samples has advanced slowly, partly because it was considered a pseudogene [10] and consequently left aside for years from the RNA arrays formerly used for the study of gene expression.

*ERAS*, as many other genes important in early development, seems to be implicated in cancer, as it is expressed in some cell lines derived from gastric, colorectal, pancreatic and breast cancers, as well as in neuroblastomas [8]. Mechanistically, several studies in cultured cells have shown that ERAS acts by binding and activating phosphoinositide-3 kinase (PI3K). The activation of the PIK3CA-AKT axis by ERAS can result in different outcomes, as increased proliferation, cell growth and cell motility [5,11,12]. These changes, in turn, could affect the ability for cellular reprogramming of somatic cells [13], and other processes important in cancer, such as epithelial-mesenchymal transition (EMT) and the metastasis of cancer cells, in a process at least partially mediated by the downregulation of miRNAs of the miR-200 family [12,14,15]. In addition, immunohistochemical and microarray expression analyses have revealed that ERAS is expressed in up to 45% of gastric carcinomas and in their liver metastasis [16,17,18] and in a lower percentage of breast tumors [12], although it is not known if this expression is relevant in tumorigenesis or simply a secondary effect of gene expression deregulation associated to tumor development.

The effect of the expression of ERAS in a living organism has not been studied yet. In order to further our knowledge of the role of ERAS in the physiology and tumoral transformation of the mammary gland, we have forced the expression of this Ras member in mammary cells of transgenic mice by means of a transgene directed by regulatory elements of keratin K5. This K5-ERAS transgene is expressed in myoepithelial basal cells of the mammary glands and other exocrine glands, as well as in basal cells of stratified epithelia. Here we show the macroscopic phenotypic effects caused by ERAS expression in keratin K5-positive cells and study the effects of ERAS deregulated expression over mammary gland physiopathology. We have found that the presence of ERAS leads to the overgrowth of epithelial tissues and to alterations in the development and function of mammary glands, giving rise to the cancerous transformation of mammary epithelia with high penetrance. ERAS causes the activation of proliferative pathways, morphological modifications in the myoepithelial cells and alterations in the expression of adhesion molecules characteristic of myoepithelial cells. Altogether, these results demonstrate the oncogenic nature of ERAS in mammary and other epithelial cells, highlight the importance of ERAS in epithelial pathophysiology, and open up the possibility that alterations in the expression of ERAS could be the causative agent of some of the syndromes of the PIK3CA-related overgrowth spectrum or of some RASopathies with unknown genetic cause.

## 2. Materials and Methods

### 2.1. Mice, Genotyping and Southern Blot

All mice husbandry and experimental procedures were performed according to European and Spanish laws and regulations and were approved by the local Animal Ethical Committee and the competent authority (code PROEX087/15). The HA-tagged human *ERAS* gene [12] was placed under the control of a 5.2 kb-upstream fragment of bovine keratin K5 promoter and a rabbit β-globin intron [19]. Transgenic mice were generated by microinjection of this construct into C57BL/6J × DBA/2J F2 (B6D2F2) embryos using standard techniques [20]. Transgenic lines were maintained by crossing transgenic mice with B6D2F1 mice. The mice were genotyped by a PCR analysis of tail genomic DNA using primers specific for the rabbit β-globin intron, as described in [21]. Alternatively, the mice genotype was assigned based on their phenotypic alterations, and confirmed by PCR once these mice were used in experiments and sacrificed. Non-transgenic littermates or B6D2F1 mice were used as control animals. A Southern blot analysis was performed using standard techniques. Transgenic copy numbers were estimated by densitometric analyses of the bands of 1.2 Kbp in EcoRI Southern and the bands of 792 and 976–982 base pairs in the PstI Southern blot (Figure S1a,b) and simple linear regression against the values obtained for the lanes loaded with the DNA of a non-transgenic mouse plus K5-ERAS plasmid in an amount equivalent to 1 and 10 transgenic copies per diploid genome. Quantitative PCR analyses of genomic DNA also confirmed that the transgenic lines carried similar copy numbers (Figure S1c).

### 2.2. Ear Surface Measurement and Organ Weighing

The mice were sacrificed and the ears carefully dissected. Then, the ears were placed avoiding any creases between two slides, and the surface of each auricle was carefully drawn in the slide and photographed including a ruler. Ear surfaces were measured by using Image J. For organ weighing, the weight of each organ—thymus, heart and the right kidney—was recorded and represented as the percentage of animal weight in 4 WT and 4 K5-ERAS male mice at the age of 10 weeks; in this way, the differences in organ weight are not due to differences in the global size of the animal. For tail skin, the 4-cm proximal portion of the skin was dissected, weighted and represented as the percentage of animal weight.

### 2.3. Whole Mount (Carmine Alum) Staining of Mammary Glands

For whole mount preparations, inguinal (number 4) mammary glands were fixed in Carnoy’s fixative and stained in carmine alum, following the protocols described in https://www2.lbl.gov/LBL-Programs/lifesciences/BissellLab/protocols.html, accessed on 20 October 2021. For quantification, the length of the colonized gland related to the total length of the gland was calculated.

### 2.4. Histology and Immunohistochemistry

Mouse tissues were dissected and immediately fixed in 10% buffered formalin or 70% ethanol and embedded in paraffin. Sections with a thickness of 5 μm were used for H&E staining or immunohistochemical preparations. The primary antibodies used are listed in Table S1. The sections were then incubated with biotinylated secondary antibodies (Jackson Immunoresearch Laboratory, Ely, UK). Immunoreactivity was revealed using the ABC-peroxidase system and the DAB substrate kit (Vector Laboratories; Burlingame, CA, USA), and the sections were counterstained with hematoxylin. The control experiments without the primary antibody gave no signals.

### 2.5. Western Blot Analysis

Whole-cell protein extracts were obtained from the mammary glands of virgin, 17.5-day pregnant female mice or from mammary tumors using standard techniques. The protein content was determined by the Bradford colorimetric protein assay (Bio-Rad Laboratories, Hercules, CA, USA). Total protein extracts (40 μg) were subjected to SDS/PAGE. The separated proteins were transferred to Amersham nitrocellulose membranes and probed with the antibodies listed in Table S1.

The blots were visualized with the Clarity Western ECL substrate (Bio-Rad, Hercules, CA, USA) using a ChemiDoc MP System and the ImageLab software (Bio-Rad, Hercules, CA, USA).

### 2.6. RNA Isolation and Quantitative RT-qPCR

Total RNA was isolated from the mammary glands of virgin, 17.5-day pregnant female mice or from mammary tumors by using miRNeasy Mini Kit (Qiagen, Hilden, Germany) according to the manufacturer’s instructions. The reverse transcription reaction was performed by using the High Capacity cDNA Reverse Transcription Kit (Applied Biosystems, Waltham, MA, USA). A quantitative RT-qPCR was performed in a 7500 Fast Real Time PCR System by using GoTaq qPCR Master Mix (Promega, Madison, WI, USA). The sequences of the oligonucleotides used are shown in Table S2. The geometrical mean of *Tbp*, *Hprt* and *Gusb* was used for gene expression normalization. Fold changes were calculated using the formula 2^−(∆CtA−∆CtB)^, where ∆CtA corresponds to the normalized expression level of K5-ERAS mammary glands or K5-ERAS tumors and ∆CtB to the normalized expression level of WT mammary glands [22].

### 2.7. Flow Cytometry Studies of Mammary Glands

Mammary gland cells from K5-ERAS and wild type mice (non-transgenic littermates or B6D2F1 mice) were harvested and analyzed as described in [23], but including 1 μg/mL of DNAse I in the digestion medium and skipping the inclusion of dispase. We studied in cell suspensions lacking lineage markers (i.e., cells negative for the hematopoietic marker CD45, the erythroid marker TER119 and the endothelial marker CD31) the distribution of luminal, myoepithelial and stromal populations in WT and K5-ERAS mammary glands by CD24 and CD29 staining, as described in [24]. The references of the antibodies used are shown in Table S1.

## 3. Results

### 3.1. ERAS Expression in K5-Positive Cells Causes Gross Phenotypic Alterations

**Figure 1:**
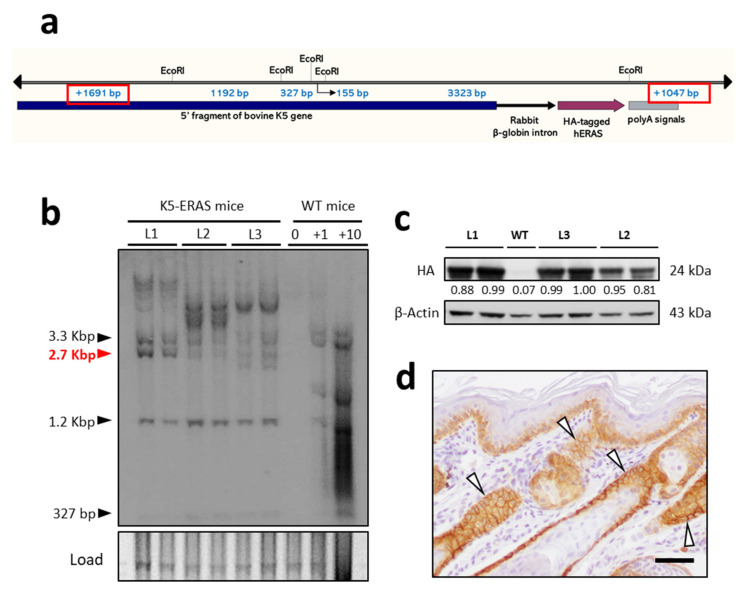
Genetic characterization and transgene expression in K5-ERAS lines. (**a**) Schematic representation of the K5-ERAS transgene structure. The location of EcoRI sites and the expected fragments in a Southern blot analysis are shown. The junction of 5′- and 3′-terminal fragments (sizes framed in red) would result in a fragment of approximately 2.7 Kbp in the case of head-to-tail tandem insertion. (**b**) Southern blot analysis of EcoRI-digested tail DNA from two mice of each line—L1, L2 and L3—and from WT mice (lane marked 0) and WT mice with the addition of the K5-ERAS plasmid insert used to obtain the transgenic mice in amounts corresponding to 1 or 10 copies per diploid genome (lanes marked +1 and +10). The approximate mobility of each band is indicated on the left; the 2.7 Kbp band resulting from head-to-tail junctions between adjacent copies is indicated in red. As a loading control, the prominent 1.3 Kbp fragment observed in the ethidium bromide staining of the EcoRI-digested mouse DNA is shown. (**c**) Western blot analysis of ERAS expression in tail skin of transgenic mice. An antibody recognizing the HA epitope was used. β-Actin was used as a loading control. (**d**) Immunohistochemical staining of tail skin of a K5-ERAS transgenic mouse with an antibody recognizing the HA epitope. ERAS expression is detected in epidermal basal cells and in the outer root sheath of hair follicles, mimicking the expression pattern at the cellular level of keratin K5. Note that the staining is stronger in the cell membrane, which is suggestive of the functionality of transgenic ERAS. Bar: 50 μm.

Previously, we have determined that ERAS expression leads to the development of several tumoral features in the non-transformed mammary cell line MCF10A [12]. With the aim of studying in depth the effects of ERAS expression in a more physiological mammary gland model, we generated transgenic mice expressing the human ERAS gene under the control of keratin K5 regulatory sequences (Figure 1a). Transgenes directed by these K5-derived regulatory sequences are consistently expressed in basal cells of stratified epithelia and in myoepithelial cells of exocrine glands, including mammary glands [21,25]. We obtained five founder mice and were able to derive transgenic lines from three of them (lines L1, L2 and L3). These lines carried a low number of copies (estimated in 3–4 copies) in a head-to-tail integration (Figure 1b and Figure S1a,b), expressed approximately similar amounts of transgenic protein (Figure 1c) and showed comparable phenotypes; so, they were used indistinctly in this article. The two other transgenic mice generated carried a higher copy number (Figure S1c) and died prematurely, being unable to transmit the transgene. Cell-type specific expression of the transgene was confirmed by immunohistochemical staining with antibodies directed against ERAS (Figure S2) or against the HA-epitope included in the transgenic construct (see the staining in the basal epidermal cells and in the outer root sheath of the hair follicles in Figure 1d and Figure S2); as this HA antibody performed better in our hands in immunohistochemistry than the ERAS antibody (see Figure S2), we used it along this study. The K5-ERAS transgene was expressed in a pattern similar to keratin K5 (Figure S3). Interestingly, the staining was detected mainly associated to membranes (arrowheads in Figure 1d), suggesting that transgenic ERAS is capable of performing its functions, as it is located in its expected subcellular location.

**Figure 2:**
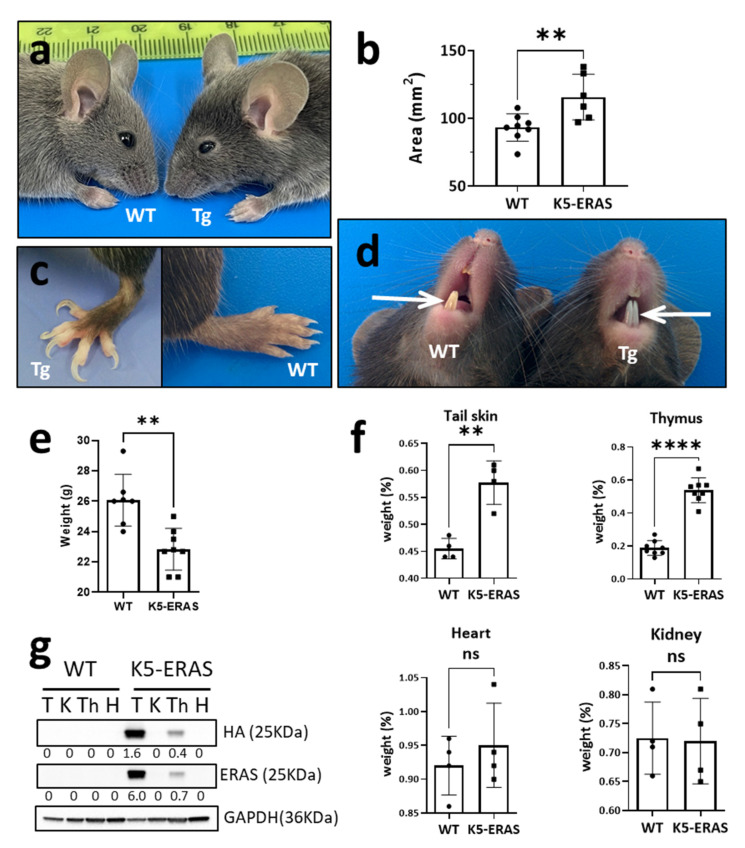
Gross phenotypic characterization of K5-ERAS transgenic lines. (**a**) Photograph of a K5-ERAS mouse (right) and a non-transgenic littermate (left) at the age of 40 days; note the increased ear size in the transgenic mouse. (**b**) Ear auricle area in 40-day-old mice. ** *p* < 0.01. (**c**) Differences in the nails of 3-month-old transgenic (left) and WT mouse (right). (**d**) Gross alterations in the color of the incisors in K5-ERAS transgenic mice. Note the whiter appearance of the upper and lower incisors (arrows) in the Tg mouse (right) compared to the WT mouse (left). (**e**) Decreased body weight of transgenic mice. The mean and S.D. of the weight of eight 60-day-old WT and Tg male mice are shown. ** *p* < 0.01. (**f**) Transgenic mice showed an overgrowth of organs and structures expressing the transgene (as skin and thymus), but not of organs without K5 expression, as the heart or the kidney. Graphs represent the weight of each organ as a percentage of the weight of each mouse. ns: non-significant differences; ** *p* < 0.01; **** *p* < 0.0001. (**g**) Western blot analyses of the tissues studied in (**f**), demonstrating K5-ERAS transgene expression in tail skin and in thymus, but nor in heart and kidney. T: tail skin; K: kidney; Th: thymus; H: heart.

K5-ERAS transgenic mice showed clear phenotypes visible to the naked eye: all of them showed big ears (Figure 2a,b) and nail overgrowth and deformity (onychogryphosis, Figure 2c), clearly visible as mice age (from 4-week-old mice onwards). In addition, their incisor teeth were whitish, in contrast with the usual yellowish color in WT mice (Figure 2d). K5-ERAS transgenic mice were smaller in size and lighter in weight than non-transgenic littermates (Figure 2e). They also showed more skin folds next to the neck (not shown), and in spite of being smaller we detected a statistically significant overgrowth of structures with transgene expression, as tail skin or thymus, but not of organs non-expressing the transgene, as the heart or the kidney (Figure 2f,g).

From these results, we conclude that the ERAS transgene is expressed with the expected tissue and subcellular specificity and that its expression causes, among other phenotypes, the overgrowth of epithelial structures.

### 3.2. Expression of K5-ERAS Transgene in Myoepithelial Cells of Mammary Glands Alters Their Development and Function

**Figure 3:**
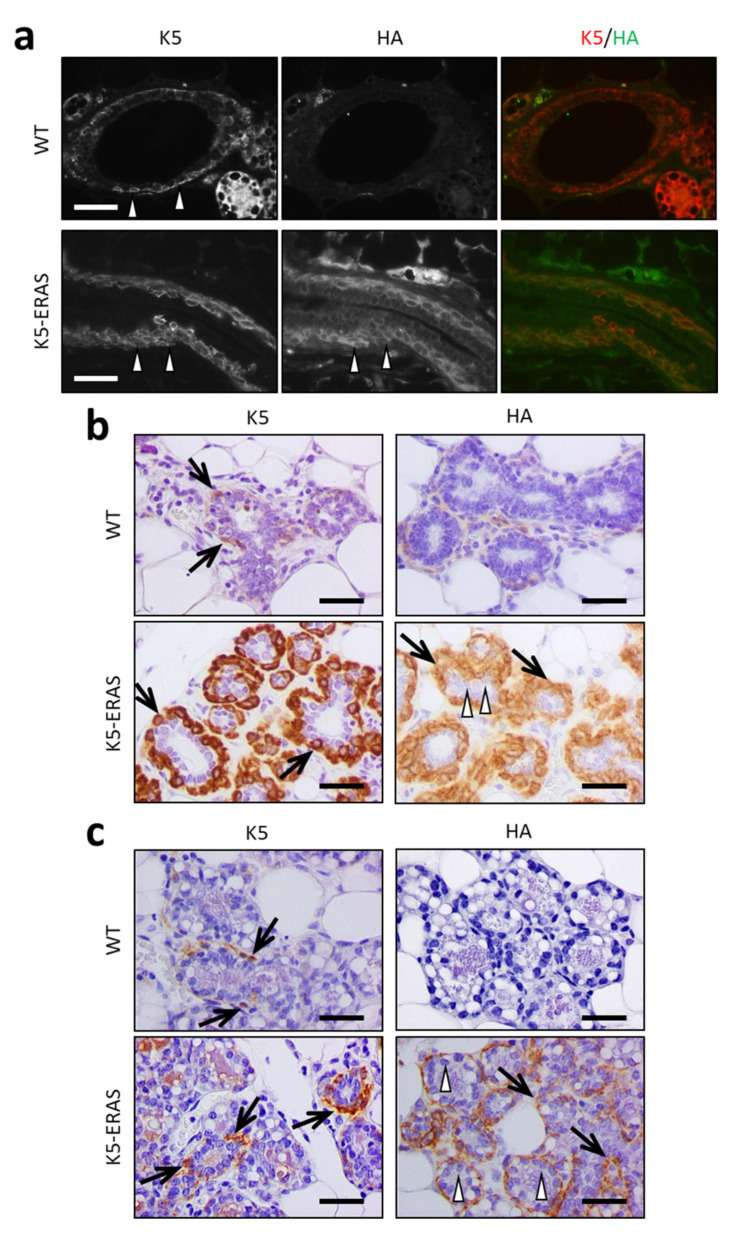
Mammary gland cell-type specific K5-ERAS transgene expression. (**a**) Immunofluorescence analysis of the expression of keratin K5 and the HA epitope (indicative of ERAS expression) in ducts from mammary glands of 10-week-old virgin animals. The genotype of the animals and the antibodies used are indicated. Note the lack of staining for the HA antibody in the mammary ducts of WT mice and the expression of both K5 and ERAS in most of the myoepithelial cells in the ducts of transgenic mice. The arrowheads point to representative K5-positive basal myoepithelial cells. (**b**,**c**) Immunohistochemical analyses of K5 and ERAS (HA epitope) expression in mammary acini of 7-month-old virgin animals (**b**) and of 17.5-day-pregnant females (**c**). The arrows point to myoepithelial cells, and the arrowheads to luminal cells. Note the co-localization of both K5 and ERAS expression in the myoepithelial cells around the acini (arrows), in contrast to the lack of expression in acinar cells (arrowheads). Myoepithelial cells in K5-ERAS females showed premalignant dysplastic changes characterized by nucleomegaly and a loss of polarity of these larger nuclei, which appeared round and perpendicular to the basal membrane of the acini in comparison to the tiny, flat nucleus disposed in parallel to the basal membrane in wild type females. Bar: 50 μm.

We next studied the expression and effect of the K5-ERAS transgene in mammary glands. In virgin 10-week-old K5-ERAS female mice, the immunofluorescent detection of the HA epitope resulted in a co-localization at the cellular level of ERAS with Keratin K5 in myoepithelial basal ductal mammary cells of (Figure 3a, arrowheads). ERAS is also expressed in basal myoepithelial alveolar cells that also express K5, both in virgin and in pregnant females (arrows in Figure 3b,c). By contrast, luminal cells do not express the transgene (arrowheads in Figure 3b,c). Strikingly, transgenic mammary glands displayed a hyperplasia of K5-positive cells (Figure 3b,c). The quantification of the area expressing K5 in nine different microscopic fields for each genotype gave values of 24.2% (SD = 10.8) for WT and 55.9% (SD = 12.2) for K5-ERAS at the age of 7 months (*p* < 0.0001). Transgenic mammary glands also showed a moderate dysplasia of these myoepithelial cells, that appeared with a variable cellular size, more rounded by the increased size of the nucleus (nucleomegaly) and with a looser appearance by the loss of nuclear polarity (Figure 3b and Figure S4).

**Figure 4:**
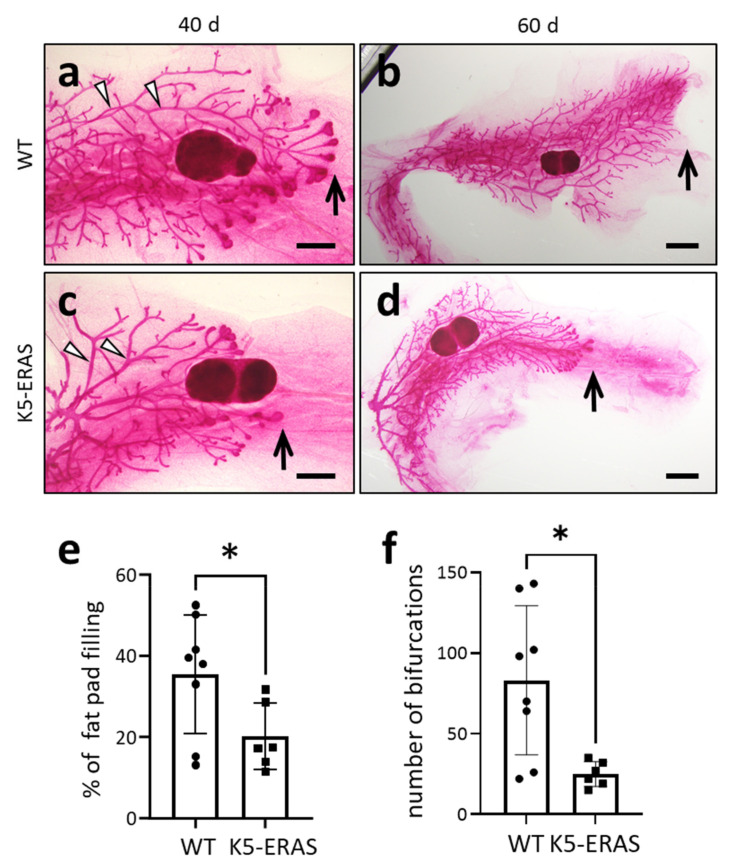
Delayed development of K5-ERAS mammary glands. (**a**–**d**) Representative images of whole mount carmine alum staining of the 4th mammary glands of WT (**a**,**b**) and K5-ERAS (**c**,**d**) mice of 40- (**a**,**c**) and 60-day-old (**b**,**d**) female mice. Note that the terminal end buds overtook the lymph nodes (the darker, round, dense islet in the center of the images) in WT (arrow in (**a**)) and not in 40-day-old K5-ERAS females (arrow in (**c**)). Note that the mammary ducts were wider in K5-ERAS (arrowheads in (**c**)) than in WT females (arrowheads in (**a**)). In 60-day-old females, the maturation delay in K5-ERAS females persisted, since the ductal tree did not colonize the whole fat pad (arrow in (**d**)) as in WTs (arrow in (**b**)). These results were obtained studying groups of 6 WT and 5 K5-ERAS 40-day-old mice and 3 WT and 3 K5-ERAS 60-day-old mice. (**e**) Quantification of fat pad colonization by mammary epithelia in WT (*n* = 4 mice) and K5-ERAS (*n* = 3) 40-day-old female mice. (**f**) Quantification of the number of bifurcations found in the epithelial tree in 40-day-old mice * *p* < 0.05. Bar: 1 mm in (**a**,**c**), and 2 mm in (**b**,**d**). The arrowheads point to the mammary ducts.

The mammary gland is an organ that is unique in its postnatal development, caused by hormonal stimuli since puberty. K5-ERAS transgenic mice showed a delay in mammary gland maturation: while in 40-day-old WT female mice the terminal end buds had overtaken the lymph node, this was not the case in K5-ERAS females (arrows in Figure 4a,c). This developmental delay can be detected both when the percentage of the gland colonized by the epithelium or the number of bifurcations of the epithelial tree are measured (Figure 4e,f). The delay remained detectable at the age of 60 days, when the ductal tree had colonized the entire fat pad in WT mice, and this colonization was still incomplete in K5-ERAS mammary glands (arrows in Figure 4b,d). This developmental delay could be due to the direct effect of ERAS on mammary cells, or, alternatively, to an indirect effect caused by the smaller size of the K5-ERAS mice (Figure 2e). Another alteration observed in these studies was the wider ductal thickness in K5-ERAS mammary glands (see arrowheads in Figure 4a,c).

**Figure 5:**
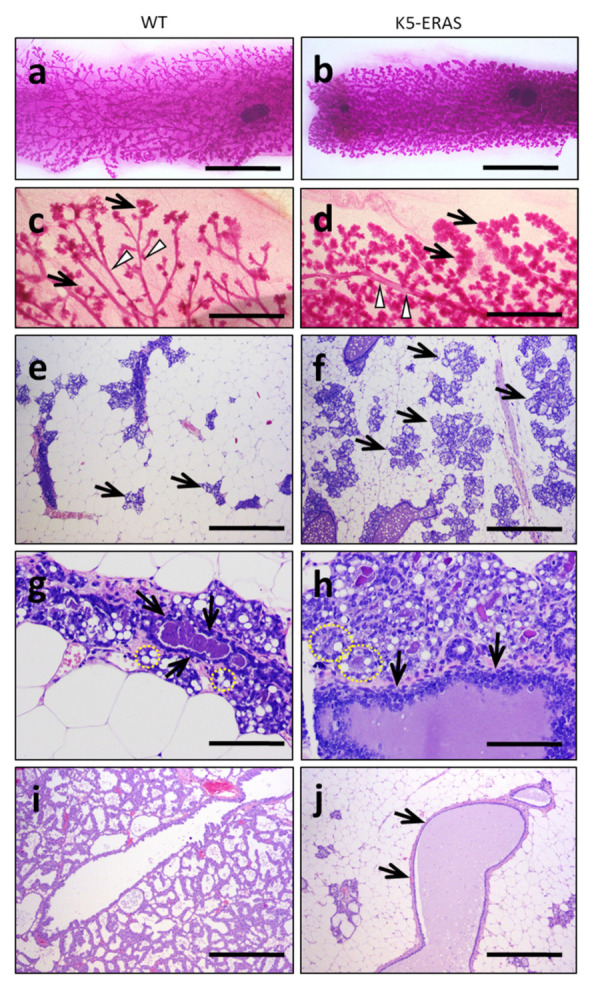
Morphological alterations in K5-ERAS mammary glands in pregnancy and lactation. (**a**–**d**) Whole mount carmine alum staining of mammary glands from WT (**a**,**c**) and K5-ERAS (**b**,**d**) female mice at a pregnancy age of 16.5 days. (**e**–**h**) H&E staining of WT (**e**,**g**) and K5-ERAS (**f**,**h**) mammary glands of 16.5 day-pregnant female mice. (**i**,**j**) H&E staining of lactating mammary glands of a WT (**i**) and a K5-ERAS female mouse (**j**). Note the larger gross size of the acini (arrows) and ducts (arrowheads) in K5-ERAS pregnant females (**d**) in comparison to WT (**c**). Microscopically, the acini in K5-ERAS pregnant females occupied an extension of the fat pad that is double that of the WT (arrows in (**e**,**f**), respectively). See, in h, mid-transversal sections of two acini (dotted yellow circles), with a size that is double that of the equivalent sections in the WT (dotted yellow circles in (**g**)). Mammary ducts in K5-ERAS pregnant females showed a larger size and hyperplasia of the epithelium (arrows in h) in comparison to WT (**g**). In day 13 of lactation, the premature regression of the acini and the cystic dilatation of ducts by milk retention in K5-ERAS females (arrows in (**j**)) were evident in comparison to WT (**i**). Bar: 7 mm in (**a**,**b**); 2 mm in (**c**,**d**); 500 μm in (**e**,**f**,**i**,**j**); 100 μm in (**g**,**h**).

We also found alterations due to ERAS expression in 16.5-day pregnant females, with a substantially larger dense acinar size in transgenic females perceptible both in the whole mount staining of mammary glands and in the H&E staining of histological slides (Figure 5a–f, arrows); in this reproductive state, the increase in duct diameter is also evident (arrowheads in Figure 5c,d). Histologic preparations of these mammary glands also revealed the larger size of acini (dotted yellow lines in Figure 5g,h) as well as ductal hypertrophy in transgenic females that also showed hyperplasia of the epithelium (arrows in Figure 5g,h). At day 13 of lactation, the complete development of lactating acini in WT females contrasts with the disabled mammary gland found in K5-ERAS females (Figure 5i,j); transgenic glands usually showed dilated ducts by milk retention and stasis inside them (arrows in Figure 5j), and a precocious involution of the mammary glands, suggesting a malfunction of myoepithelial cells to contract and squeeze the acini and ducts to eliminate milk. This phenotype is probably the cause of the very low fitness observed for female K5-ERAS mice in breeding newborn mice (not shown).

In sum, an inappropriate ERAS expression in mammary glands leads to the hyperplasia and dysplasia of myoepithelial mammary cells, to a delay in postnatal mammary development and to malfunctioning myoepithelial cells in lactating females.

### 3.3. ERAS Expression Causes Spontaneous Tumor Development

**Figure 6:**
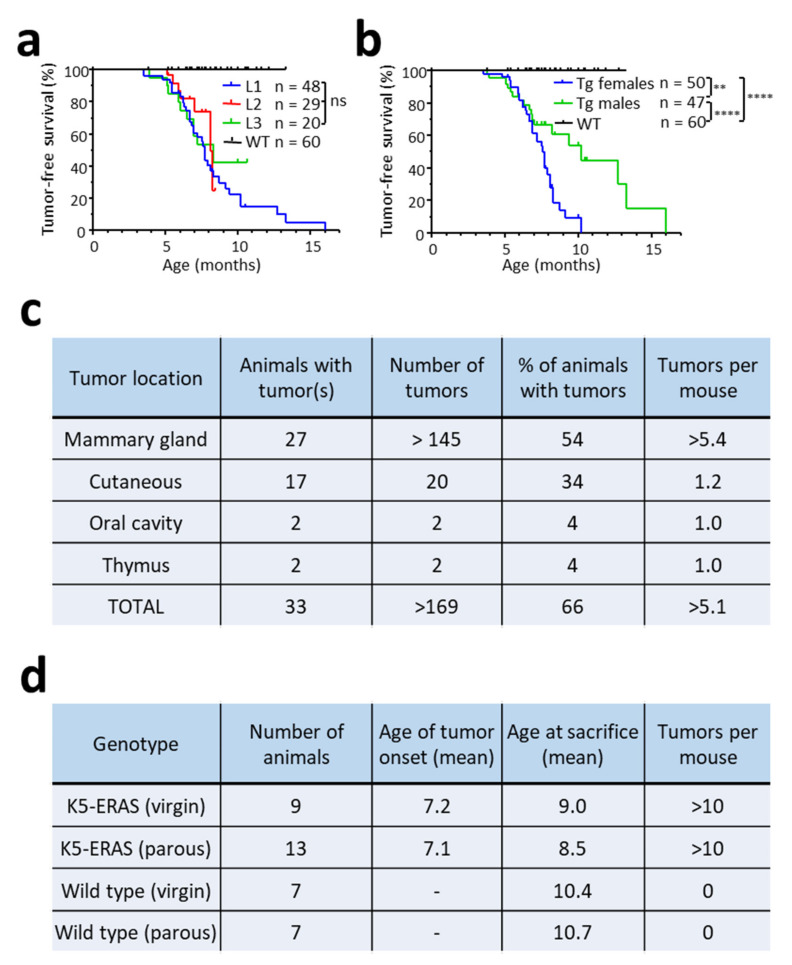
Spontaneous tumorigenesis in K5-ERAS transgenic mice. (**a**) Kaplan–Meier analysis of tumor-free survival in populations of wild type and transgenic mice from L1, L2 and L3 lines. This plot includes all the tumor types observed. The number of mice studied was 60 WT mice and 48, 29 and 20 transgenic mice form L1, L2 and L3 lines, respectively. The differences between transgenic lines are non-significant. (**b**) Tumor incidence in female and male K5-ERAS transgenic mice. ** *p* < 0.01; **** *p* < 0.0001. (**c**) Location of the tumors found in the group of female transgenic mice studied longitudinally. (**d**) Mammary tumorigenesis in multiparous and virgin K5-ERAS and WT females. Neither tumor onset nor multiplicity were affected by pregnancy.

We studied whether ERAS expression renders the mice more prone to tumor development, with a special emphasis in mammary glands. With this aim, we followed the spontaneous development of tumors in populations of 60 WT mice and 97 mice of L1, L2 or L3 K5-ERAS transgenic lines, as indicated in Figure 6a. At around 5 months of age, the three lines of transgenic mice began to develop tumors at a similar pace, while WT mice did not develop any (Figure 6a). The tumorigenesis rate in female transgenic mice was significantly faster than in male transgenic mice, due to the appearance of numerous fast-growing mammary gland tumors (Figure 6b). Figure 6c shows the tumor distribution in the female group; the most frequent tumor type was mammary gland carcinoma, with more than 50% of the animals developing fast-growing mammary tumors throughout their life. In addition, many of these animals showed multicentric tumors that affected several mammary glands, making it difficult to accurately count the number of tumors (Figure 6c). Mammary tumor formation does not seem to be affected by pregnancy, as both tumor multiplicity and the time of tumor formation were similar in virgin and multiparous transgenic females (Figure 6d). Tumors were also found in other locations expressing the transgene, such as skin, oral epithelia and thymus, although with less frequency and multiplicity. Altogether, these results indicate that ERAS expression, in an otherwise normal cellular environment, is enough for cell transformation, both in mammary cells and in cells of other epithelia expressing the transgene. No lung metastases were observed in 19 K5-ERAS mice with tumors (15 females and 4 males), probably due to the fast growth of tumors.

### 3.4. Mammary Tumors in K5-ERAS Mice Are Multicentric and of Myoepithelial Origin, Similar to Human Adenomyoepitheliomas

**Figure 7:**
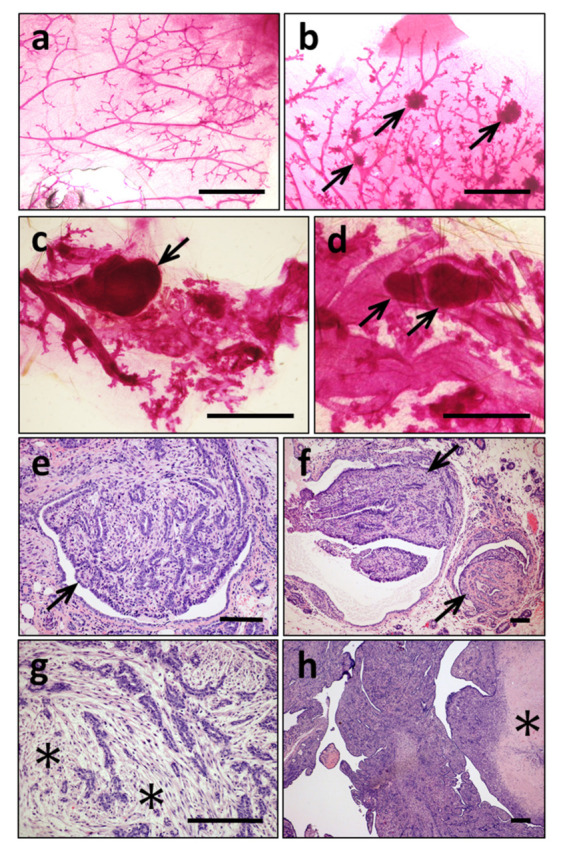
Gross and histological analysis of mammary gland tumors in K5-ERAS female mice. (**a**–**d**) Whole mount carmine alum staining of mammary glands of WT mice (**a**) and of K5-ERAS mice (**b**–**d**); K5-ERAS samples show multiple tumors associated to epithelial ducts and acini (arrows in **b**–**d**). In (**c**) and (**d**), larger tumoral structures are seen growing inside dilated mammary ducts. (**e**–**h**) Representative histological features of mammary tumors in K5-ERAS female mice. (**e**,**f**) Intraductal carcinomas with overgrowth of myoepithelial cells (myoepitheliomas). (**g**,**h**) Larger myoepitheliomas showed extensive stromal hyalinization (asterisks in (**g**)) or necrotic regions (asterisk in (**h**)). Bar: 2 mm in (**a**–**c**); 1 mm in (**d**) and 100 μm in (**e**–**h**).

The whole mount staining of mammary glands revealed the multicentric nature of these tumors (arrows in Figure 7b) and the presence of dilated ducts containing tumoral lesions resembling ductal carcinomas (arrows in Figure 7c,d). Histologically, these tumors presented a papillary-tubular pattern (Figure 7e,f), and the presence of multiple nodular carcinomas filling the mammary ducts was confirmed (Figure 7f, arrows). 

The majority of mammary tumors developed in K5-ERAS female mice were quite homogeneous, with disorganized myoepithelial growth and dysplastic epithelial cells showing moderate pleomorphism. Frequently, mammary tumors presented abundant hyalinization of the stroma (Figure 7g, asterisks). The fast development of these tumors leads to the presence of necrotic regions in the central part of large tumors (Figure 7h, asterisk).

**Figure 8:**
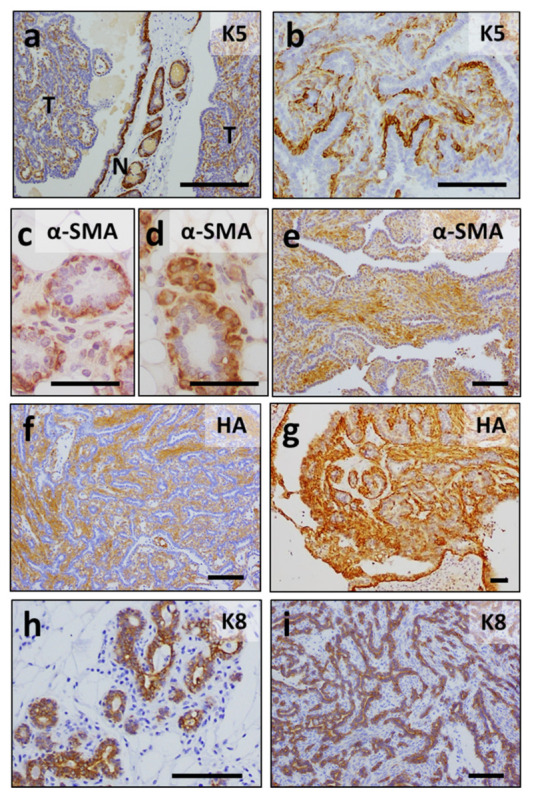
Immunohistochemical analyses of K5-ERAS mammary gland tumors. (**a**–**i**) Immunohistochemical staining of non-tumoral mammary gland ((**c**,**d**,**h**), and region marked N in (**a**)), tumoral samples ((**b**,**e**–**g**,**i**) and regions marked T in (**a**)) of K5-ERAS transgenic (**a**,**b**,**d**–**g**,**i**) and non-tumoral sample of 7-month-old WT mice (**c**,**h**) using antibodies specific for the indicated epitopes. Note the expression of K5 (**a**,**b**) and α-SMA (**e**) in K5-ERAS tumors, confirming their myoepithelial origin, as well as the co-localized expression of ERAS (HA epitope) (**f**,**g**), while acinar cells expressed K8 (**i**). Bar: 100 μm except for (**c**,**d**) (50 μm).

A immunohistochemical characterization of these tumors revealed that they expressed keratin K5 (although frequently to a lesser extent than non-tumoral mammary tissue, Figure 8a,b), and α-SMA, similar to the myoepithelial cells of both WT and K5-ERAS (Figure 8c–e), confirming their myoepithelial origin. Tumoral samples also expressed the K5-ERAS transgene, as stated by the HA-positive staining of these tumors (Figure 8f,g). The cuboidal epithelial cells of the acini lined by the myoepithelial component of the tumors were positive for K8 expression, in a pattern equivalent to K8 expression in non-tumoral WT glands (Figure 8h,i). We also studied the expression of the estrogen receptor ESR1, in order to determine if tumor growth could be driven by a persistent ESR1 expression. We found that the expression of ESR1 in normal mammary tissue from WT and K5-ERAS mice was similar to that found in tumoral transgenic tissue (Figure S5), suggesting that tumoral growth in K5-ERAS mammary glands is non-related to ESR1 upregulation. Both the histological appearance of the tumors and the expression of differentiation markers allow for the classification of this tumor type as similar to human malignant adenomyoepithelioma, a rare human breast cancer type [26,27,28].

In addition to mammary gland tumor development, we found that 12 out of 97 K5-ERAS females had developed supernumerary nipples (a condition named polythelia). These extra nipples, when present, appeared in the inguinal region close to the bona fide nipples of the 4th mammary pair (Figure S6a,b). We also found alterations in nipple epithelia in seven of the animals, with hyperplasia and hyperkeratosis, as well as development of ductal carcinomas (Figure S6d, arrow) and sebaceous adenomas (Figure S6e) filling the mammary sinuses. Interestingly, we also found occasional mammary tumors in K5-ERAS transgenic male mice histologically similar to those described for female mice (not shown). Therefore, ERAS has a strong transforming ability, being able to produce mammary tumors in the small rudiment of mammary tissue present in male mammary glands [29].

### 3.5. K5-ERAS Mammary Glands and Tumors Showed Increased Activity of Proliferative Pathways

**Figure 9:**
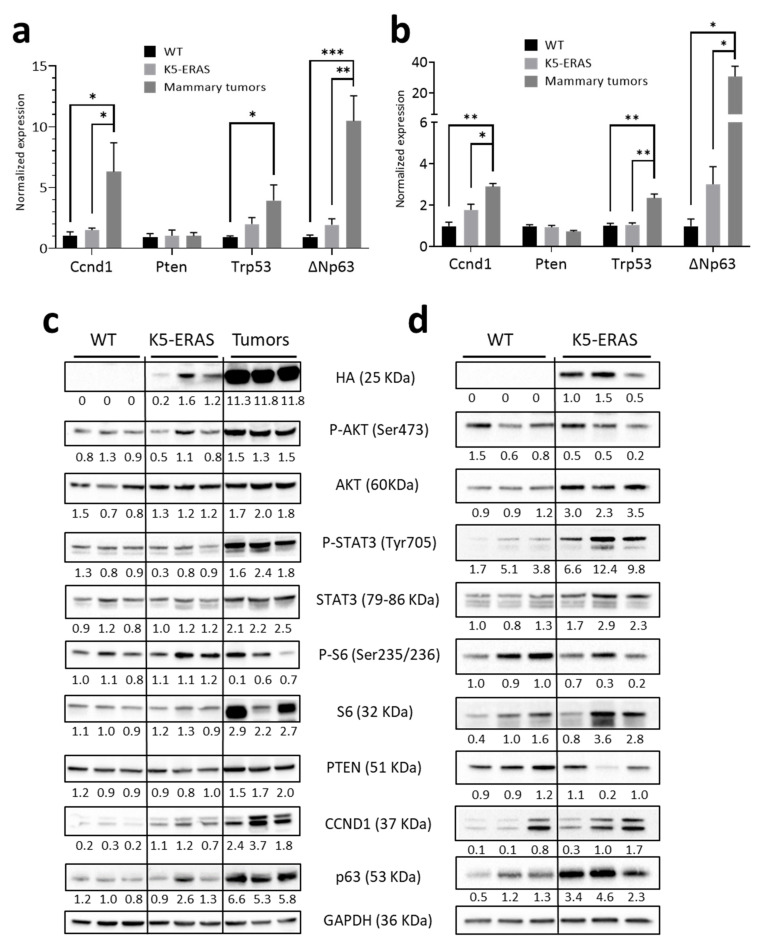
Biochemical characterization of K5-ERAS mammary glands and tumors. (**a**,**b**) RT-qPCR analyses of the expression of genes implicated in proliferation and tumoral transformation in mammary glands of 17.5-day pregnant WT and K5-ERAS mice, as well as in K5-ERAS mammary tumors (**a**) and in virgin mammary glands (**b**). (**c**) Western blot analysis of three different samples of mammary glands from 17.5-day pregnant WT and K5-ERAS mice, and of K5-ERAS mammary tumors. (**d**) Similar study in mammary glands from virgin animals. In (**c**,**d**), phosphoproteins are normalized against the corresponding total proteins. We used between 3 and 5 samples of each genotype for the results included in (**a**) and three samples per genotype in (**b**). * *p* < 0.05; ** *p* < 0.01; *** *p* < 0.001.

We wondered about the biochemical changes caused in both normal and tumoral mammary glands by ERAS, and studied, in mammary glands on day 17.5 of pregnancy (in order to increase the relative amount of epithelial cells in the organ with respect to glands from virgin animals) and in K5-ERAS mammary tumors, the expression of genes related to proliferation, as Ccnd1, finding a significant increase in K5-ERAS tumors. Ccnd1 was also slightly induced in K5-ERAS WT glands, suggesting the higher proliferation ability of mammary tissue expressing ERAS (Figure 9a). We also studied the expression of the tumor suppressor genes Pten and Trp53, and of ΔNp63, a factor implicated in the maintenance of epithelial stem cells; we did not find differences in the expression of Pten, but both Trp53 and ΔNp63 were augmented in K5-ERAS transgenic mammary glands, and even more noticeably in tumoral samples. The western blot analysis confirmed the increase in expression of CCND1 in transgenic glands and even, in a stronger way, in K5-ERAS tumors (Figure 9c and Figure S8a). ERAS is known to act through the AKT pathway [5,13,14]; the western blot analysis displayed PI3K-AKT pathway activation (as indicated by the increase in the amount of the Ser473 phosphorylated active form of AKT) and an increase in the activity of the STAT3 pathway in tumoral samples (Figure 9c and Figure S8a).

The immunohistochemical analysis showed more clearly an increase in P-AKT in non-tumoral mammary glands from 17.5-day pregnant females: while WT mammary glands presented a diffuse P-AKT staining of the cytoplasm both in basal and in luminal cells in ducts and in acini, we found in K5-ERAS mammary glands a conspicuous staining of the cytoplasm of basal myoepithelial cells (Figure S7a, arrows). The IHQ also showed a clear increase in the amount of myoepithelial nuclei that stained positive for p63 both in the ducts and acini (arrows in Figure S7b,c). Given that p63 is a myoepithelial cell marker [30], these results suggest that an amplification of the basal cell compartment in K5-ERAS mammary glands is taking place.

We also performed a similar study in mammary glands from virgin animals, obtaining an increase of CCND1 and p63 in transgenic samples similar to that seen in mammary glands from pregnant animals (Figure 9b). Surprisingly, we also detected other differences between non-tumoral WT and K5-ERAS samples that could contribute to the tumoral transformation of the latter, as P-STAT3 increased in the western blots (Figure 9d and Figure S8b).

Altogether, these results indicate that ERAS causes several changes in molecules and pathways related with cell proliferation that could result in the tumoral transformation in mammary glands. They also suggest an enlargement of the compartment of myoepithelial cells.

### 3.6. Mammary Cells from Aged K5-ERAS Female Mice Show a Decreased Expression of the Adhesion Molecule CD29

CD24 and CD29 (also known as integrin beta-1) are adhesion molecules whose expression level can be used in flow cytometry studies to distinguish between the different cellular populations present in the mammary gland [24], making it possible to differentiate luminal cells (highly positive for CD24 and with medium-low positivity for CD29) from basal myoepithelial cells (positive for both CD24 and CD29) and stromal cells (low expression for CD24).

**Figure 10:**
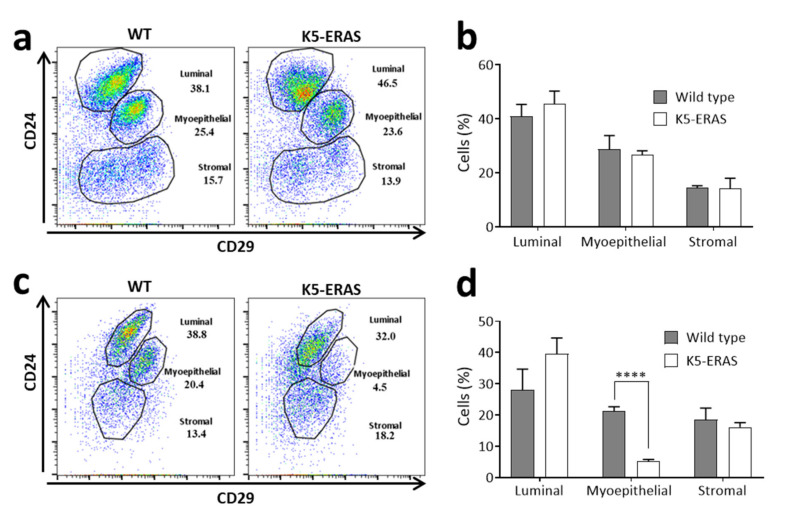
Flow cytometry study of cell-surface markers in K5-ERAS mammary cells. (**a**,**b**) Analysis of CD24 and CD29 expression in the mammary cell population negative for hematopoietic, erythroid and endothelial linage markers in 10-week-old virgin female mice. (**a**) Example of the population distribution in one WT and one K5-ERAS female mouse; (**b**) mean and standard deviation of the data obtained from four mice of each genotype. The differences observed are statistically non-significant. (**c**,**d**) The same analyses were performed in 7-month-old virgin female mice. (**c**) Representative plot of one WT and one K5-ERAS female mouse; (**d**) mean and SD of four different mice of each genotype. **** *p* < 0.0001.

With the aim of understanding whether ERAS produces any alteration in the abundance of these mammary cell populations, we studied the expression of CD24 and CD29 in lineage-negative-mammary gland cells from WT and K5-ERAS mice of several ages. In young (10-week-old) female mice, both the distribution of cell population and the percentages of luminal, myoepithelial and stromal cells were roughly the same in K5-ERAS female mice and wild type littermates (Figure 10a,b). By contrast, a similar study performed in aged (7-month-old) virgin female mice showed clear differences between genotypes in the distribution of cells in the plot (Figure 10c): while WT mice showed a distribution similar to that of young mice, in K5-ERAS mammary glands a decrease in the level of CD29 staining was evident. As a result, a displacement of these cells to the left part of the plot and a concomitant significant reduction in the percentage of cells in the myoepithelial-gated population were found (Figure 10c,d).

This result contrast with the increase in cells expressing myoepithelial cell markers observed in the keratin K5 and p63 immunohistochemistry analyses (Figure 3b,c and Figure S7b,c). From these data we conclude that, as previously described for MCF10A cells [12], ERAS expression in mammary myoepithelial cells causes a diminution in the expression of CD29; consequently, these functionally altered myoepithelial cells are found, in the CD24 and CD29 plots, outside the usual gate for myoepithelial cells.

## 4. Discussion

Several experiments performed recently by us and others indicate that ERAS is important in tumorigenesis. So, in murine mutagenesis experiments using the Sleeping Beauty transposon, which result in mammary gland tumors, melanoma and metastatic medulloblastoma, ERAS emerges as a candidate oncogene [15,31,32]. Moreover, ERAS expression has been detected in a number of tumoral cell lines [7,8,17], and we have found that it induces strong changes associated to cellular transformation in the otherwise non-tumoral human mammary MCF10A cell line [12]. Likewise, its expression in mammary tumoral cell lines leads to an aggravation of their tumoral potential [12]. In addition, MMTV-induced insertional mutagenesis experiments in mice of several genetic backgrounds have also pointed at a possible implication of ERAS in mammary tumorigenesis [33,34,35]. Finally, in patients with primary colon cancer gene fusions between *USP9X* and *ERAS* have been found, resulting in the constitutive expression of relatively high levels of ERAS [36]. Altogether, these results seem to indicate that the inappropriate expression of *ERAS*, a gene that is usually silenced except in early embryonic life, is able to induce a tumoral transformation in several organs.

In this report, we demonstrate, by expressing human ERAS in transgenic mice under the control of K5-derived regulatory sequences, the oncogenic role of ERAS in epithelial cells from mammary glands. Mice expressing ERAS in myoepithelial mammary cells developed mammary tumors with a high incidence and multiplicity. These mammary tumors typically begin as multicentric carcinomas in situ that evolve to intraductal carcinomas, with abundant myoepithelial cell growth and a variable level of hyalinization of the stroma, eventually blocking the mammary ducts. Mammary tumors in K5-ERAS mice express the estrogen receptor ESR1 but do not seem to be dependent on hormonal signaling, as transgenic females with several pregnancies develop mammary tumors with a prevalence similar to that of virgin females (Figure 6c). They usually express proteins characteristic of myoepithelial cells, as keratin K5 and Sma-1, being histologically similar to malignant adenomyoepitheliomas, a rare human tumor type of unknown etiology. Thus, the study of ERAS expression in this particular human tumor type would be of great interest, but this is a rare tumor type, and it is not easy to obtain samples.

Although ERAS does not seem to be essential for life, as ERAS knock-out mice are viable and fertile [5], our results suggest that high expression levels of ERAS are detrimental. From the transgenic founders we generated, we could only derive lines from the three mice with a lower copy number. These mice, although viable, showed several notable phenotypic alterations: firstly, they showed a whitish dental surface; a similar phenotype has been described in mutant mice lacking *Nrf2*, a transcription factor fundamental in iron metabolism [37]. Nrf2 absence in mice leads to an aberrant iron deposition in the papillary layer cells of the enamel organ and to altered ameloblasts, causing a loss of iron deposition on the enamel surface [38]. As the K5 promoter that we have used to direct the expression of ERAS is active in ameloblasts [39,40,41], it is conceivable that ERAS expression in this cell type may alter ameloblast functioning, resulting in a defective enamel iron deposition and discoloration.

Secondly, K5-ERAS transgenic mice are smaller than their non-transgenic siblings, and this phenotype does not seem to be due to feeding problems caused by malocclusion nor tumor formation in the palate, esophagus or stomach (not shown). They also showed an overgrowth of the epithelial organs that express the transgene, as nails, ears, skin and thymus (Figure 2). It is not surprising that ERAS expression (that is accompanied by the activation of the AKT pathway and other molecules leading to cell growth and division, as CCND1, —Figure 9, Figure S7 and [12]—) produces a phenotype quite similar to the overgrowth syndromes caused by the overactivation of the PIK3CA-AKT pathway, such as the Cloves, Proteus and PTEN hamartoma tumor syndromes, among others [42,43]. Interestingly, in some cases these syndromes are associated to a higher cancer predisposition, similar to what happens in K5-ERAS transgenic mice. In this context, *ERAS* adds up to the list of genes whose mutation can lead to overgrowth syndromes (as *AKT*, *PIK3CA*, *PTEN*) or RASopathies (as *H-RAS*, *RASA1*, *NF1* and *RAF1*, among others [44]).

Strikingly, K5-ERAS mice from the three lines we generated are notoriously docile (they bear the interaction with the experimenter better than WT mice, reacting in a less lively manner and making their management easier; data not shown); although we have detected a transgenic expression in the epithelia of the choroid plexus (not shown), this phenotype could also be due to the expression in other hypothetical K5-positive brain structures. It is also possible that this particular docility is the manifestation of some kind of mental disability, a deficit in the development of the central nervous system or a cognitive defect; these trends are common in a number of RASopathies, both in human patients and in mouse models [45] and could well be caused, in K5-ERAS mice, by the activation of the RAS pathway in a yet undetermined location of the central nervous system.

The K5 promoter that we have used is active in the basal layer of stratified epithelia [46] and has been widely used to direct the expression of transgenes to the skin, often resulting in tumors [47,48,49,50]. It is thus noteworthy that ERAS expression causes, above all, mammary tumors, even in male mice. It is possible that mammary glands are particularly sensitive to the inappropriate expression of ERAS, and in fact we have previously described that a small percentage of human tumors overexpress ERAS [12]. Although it is possible that the inappropriate expression of ERAS in human breast tumors is a consequence of tumor development, our finding that ERAS expression causes mammary tumors in mice strongly argues in favor of an oncogenic role in humans.

ERAS affects the mammary glands at multiple levels. We first found a delay in the post-pubertal development of the mammary glands. This delay could be the direct consequence of ERAS activity in myoepithelial cells of the terminal end bud, or it could be an indirect effect over the mammary gland due to a delay in sexual maturation caused by the decreased weight of transgenic mice (Figure 2e). To distinguish between these possibilities, more experiments would be needed.

ERAS also alters the cellular structure of the mammary ducts, making them wider (Figure 4a,c and Figure 5c,d,g,h). In aged mice, the mammary ducts even become exaggeratedly enlarged (Figure 7c,d), probably preventing the correct functioning of the gland. In fact, although there is an increase in acini formation in pregnant transgenic females in comparison to WT mice (Figure 5e,f), the mammary glands in lactating females are frequently blocked, filled with milk that cannot be released (milk stasis), and end up causing the involution of the mammary glands well before weaning. This is probably the cause of the low reproductive performance of K5-ERAS female mice, as, although the pregnancy and delivery are normal in these mice, most of the newborns die in the first days of life (independently of their transgenic status), being clearly undernourished (not shown).

At the molecular level, ERAS is known to act through the PIK3CA-AKT pathway [5,12,13,14] (involved in cell growth and proliferation, as well as in promoting tumor formation); consequently, we have found in ERAS-expressing mammary glands the activation of this PIK3CA-AKT pathway, as well as the overexpression of cyclin D1, a protein needed for the progression trough the cell cycle. In tumors, these alterations are even more accentuated. Overall, these molecular alterations render the glands more prone to tumoral transformation.

Another remarkable change caused by ERAS is the alteration in the expression of the adhesion molecules used to define the different epithelial cell types in the mammary gland (i.e., luminal and myoepithelial cells). Previous results from our laboratory revealed an inhibition of the expression of CD29 (ITGB1) in ERAS-expressing human mammary MCF10A cells [12]. In murine mammary glands the same may occur, and as a consequence, the diminished expression of CD29 that we have found in K5-ERAS myoepithelial cells makes it difficult to study this population by flow cytometry. Interestingly, the decreased expression of the adhesion molecule CD29 could be responsible for the dysplastic looser and rounded appearance of myoepithelial cells in K5-ERAS in comparison to WT female mice (Figure 3b). Whether these changes facilitate the metastatic spread of tumoral cells remains to be studied.

In summary, the results included in this report clearly indicate the oncogenic nature of ERAS in murine mammary glands, and suggest that a derepression of ERAS in human mammary gland may also act as an oncogene

## 5. Conclusions

The results reported here demonstrate that ERAS acts as a true oncogene in mammary glands and suggest that ERAS could be implicated in any of the RASopathies and/or overgrowth syndromes of unknown genetic origin. ERAS expression in mammary myoepithelial cells lead to alterations in the morphology and gene expression of these cells, and to the tumoral transformation of mammary glands with high penetrance. The tumors originated are intraductal, multicentric, fast growing and resemble malignant adenomyoepitheliomas; this makes K5-ERAS transgenic mice an excellent model to study the origin and behavior of this rare human tumor. 

## Data Availability

This statement should be excluded, as the study did not report any data.

## Supplementary Materials

The following are available online at https://www.mdpi.com/article/10.3390/cancers13215588/s1, Table S1: List of antibodies used for immunohistochemistry, immunofluorescence, western blotting and flow cytometry, Table S2: Sequences of primers used in RT-qPCR studies. Figure S1: Further genetic characterization of K5-ERAS lines, Figure S2: Immunohistochemical detection of transgene expression by antibodies specific for ERAS and HA tag in tail skin criosections, Figure S3: K5-Eras transgene is expressed in a pattern similar to Keratin 5, Figure S4: Comparison of nuclear size of myoepithelial cells in mammary glands of 17.5-day-pregnant females, Figure S5: Immunohistochemical analyses for ESR1 in K5-ERAS mammary gland tumors, Figure S6: Nipple alterations in K5-ERAS female mice, Figure S7: Immunohistochemical analysis of P-AKT (Ser473) and p63 expression in WT and K5-ERAS 17.5-day pregnant mammary glands, Figure S8: Graphical representation of the quantification of the western blots shown in Figure 9.

## Abbreviations

ERAS: Embryonic RAS; HA, hemagglutinin A; H&E, hematoxylin and eosin; Kbp: Kilo base pair; ns: non-significant differences; SD: standard deviation of the mean; Tg, transgenic; WB: western blot; WT, wild type.
