# Supplementary material for: ERAS, a Member of the Ras Superfamily, Acts as an Oncoprotein in the Mammary Gland
Source: Cancers (Basel). 2021 Nov 8;13(21):5588. doi: 10.3390/cancers13215588 (PMC8582886; doi:10.3390/cancers13215588)
Supplement: Supplementary file 1 [file cancers-13-05588-s001.zip › Supplementary Figure 1.pptx]

## Slide 1
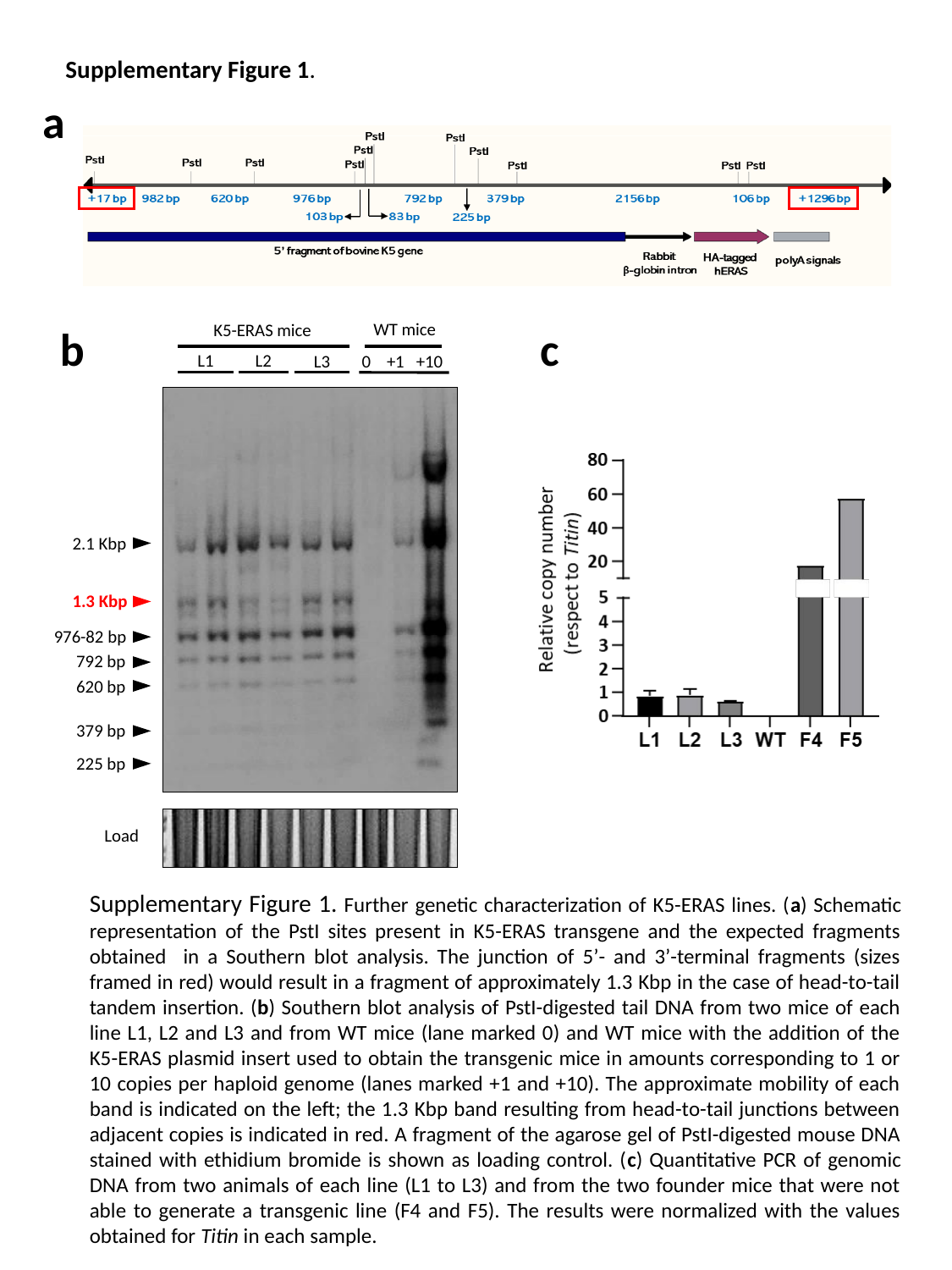

Supplementary Figure 1.
a
WT mice
K5-ERAS mice
b
L1
L2
L3
0 +1 +10
2.1 Kbp
1.3 Kbp
976-82 bp
792 bp
620 bp
379 bp
225 bp
Load
c
Supplementary Figure 1. Further genetic characterization of K5-ERAS lines. (a) Schematic representation of the PstI sites present in K5-ERAS transgene and the expected fragments obtained in a Southern blot analysis. The junction of 5’- and 3’-terminal fragments (sizes framed in red) would result in a fragment of approximately 1.3 Kbp in the case of head-to-tail tandem insertion. (b) Southern blot analysis of PstI-digested tail DNA from two mice of each line L1, L2 and L3 and from WT mice (lane marked 0) and WT mice with the addition of the K5-ERAS plasmid insert used to obtain the transgenic mice in amounts corresponding to 1 or 10 copies per haploid genome (lanes marked +1 and +10). The approximate mobility of each band is indicated on the left; the 1.3 Kbp band resulting from head-to-tail junctions between adjacent copies is indicated in red. A fragment of the agarose gel of PstI-digested mouse DNA stained with ethidium bromide is shown as loading control. (c) Quantitative PCR of genomic DNA from two animals of each line (L1 to L3) and from the two founder mice that were not able to generate a transgenic line (F4 and F5). The results were normalized with the values obtained for Titin in each sample.
