# Supplementary material for: ERAS, a Member of the Ras Superfamily, Acts as an Oncoprotein in the Mammary Gland
Source: Cancers (Basel). 2021 Nov 8;13(21):5588. doi: 10.3390/cancers13215588 (PMC8582886; doi:10.3390/cancers13215588)
Supplement: Supplementary file 1 [file cancers-13-05588-s001.zip › Supplementary Figure 2.pptx]

## Slide 1
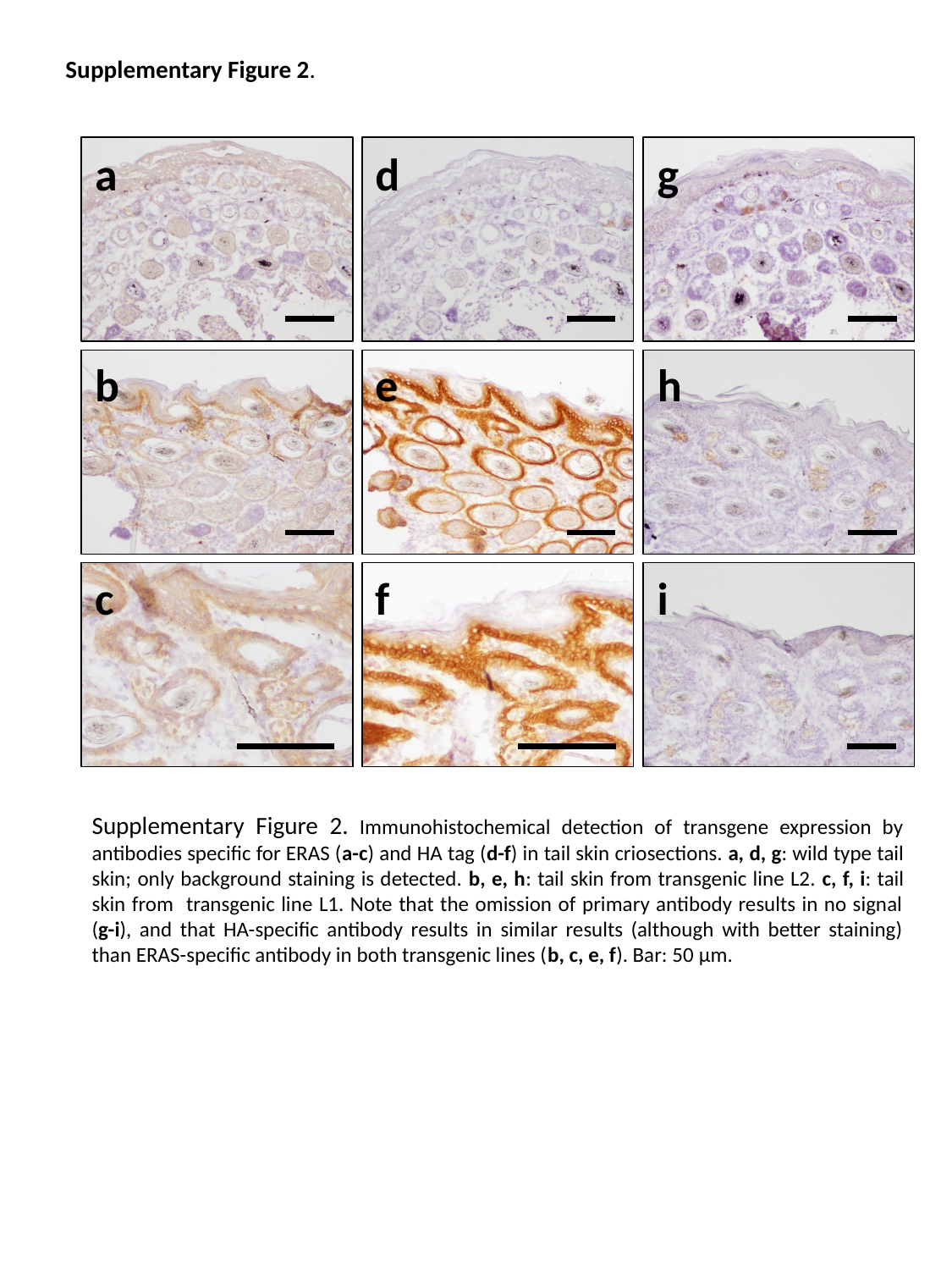

Supplementary Figure 2.
a
d
g
b
e
h
c
f
i
Supplementary Figure 2. Immunohistochemical detection of transgene expression by antibodies specific for ERAS (a-c) and HA tag (d-f) in tail skin criosections. a, d, g: wild type tail skin; only background staining is detected. b, e, h: tail skin from transgenic line L2. c, f, i: tail skin from transgenic line L1. Note that the omission of primary antibody results in no signal (g-i), and that HA-specific antibody results in similar results (although with better staining) than ERAS-specific antibody in both transgenic lines (b, c, e, f). Bar: 50 μm.
