# Supplementary material for: ERAS, a Member of the Ras Superfamily, Acts as an Oncoprotein in the Mammary Gland
Source: Cancers (Basel). 2021 Nov 8;13(21):5588. doi: 10.3390/cancers13215588 (PMC8582886; doi:10.3390/cancers13215588)
Supplement: Supplementary file 1 [file cancers-13-05588-s001.zip › Supplementary Figure 3.pptx]

## Slide 1
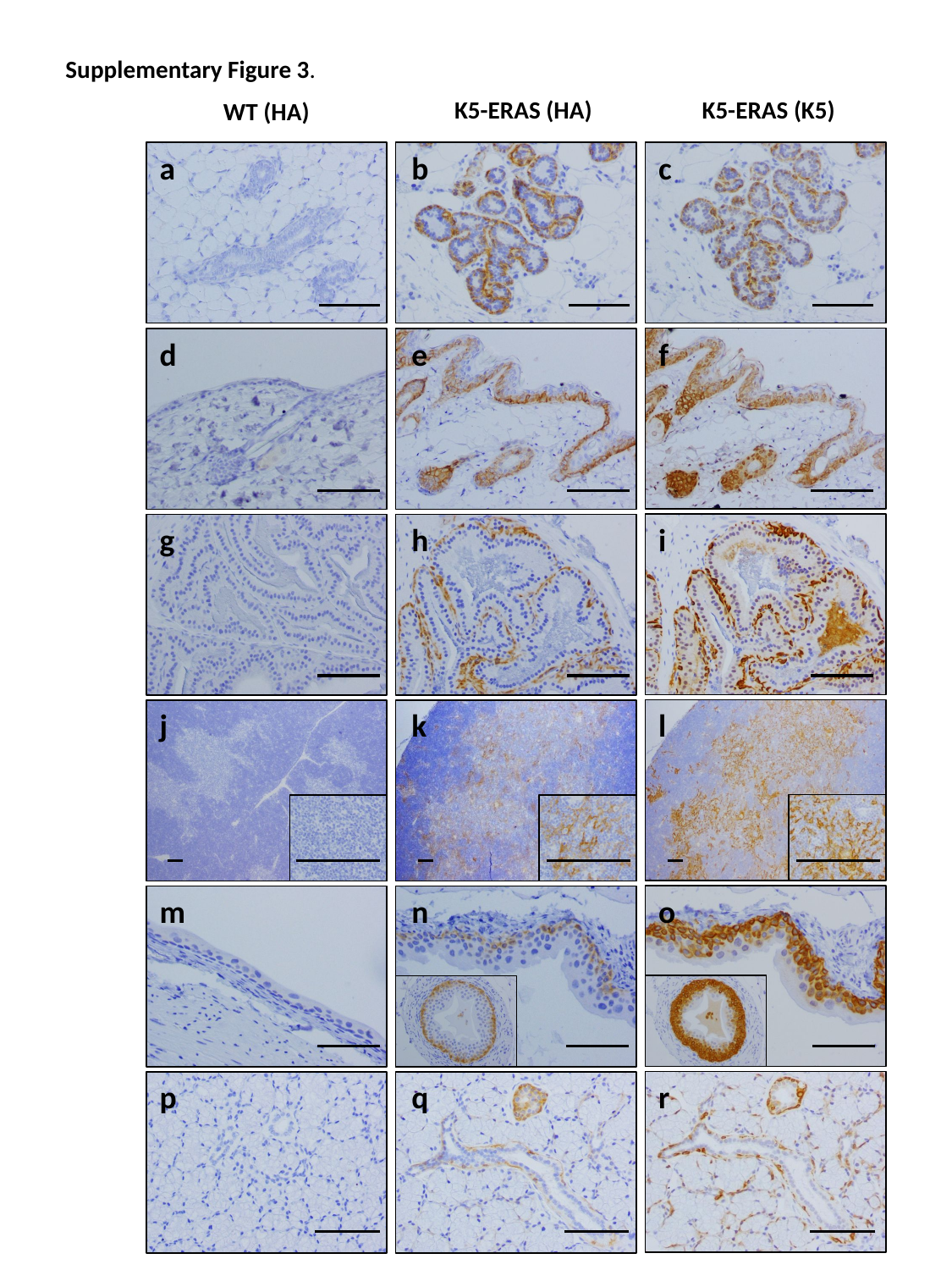

Supplementary Figure 3.
K5-ERAS (HA)
K5-ERAS (K5)
WT (HA)
a
b
c
d
e
f
g
h
i
j
k
l
m
n
o
p
q
r

## Slide 2
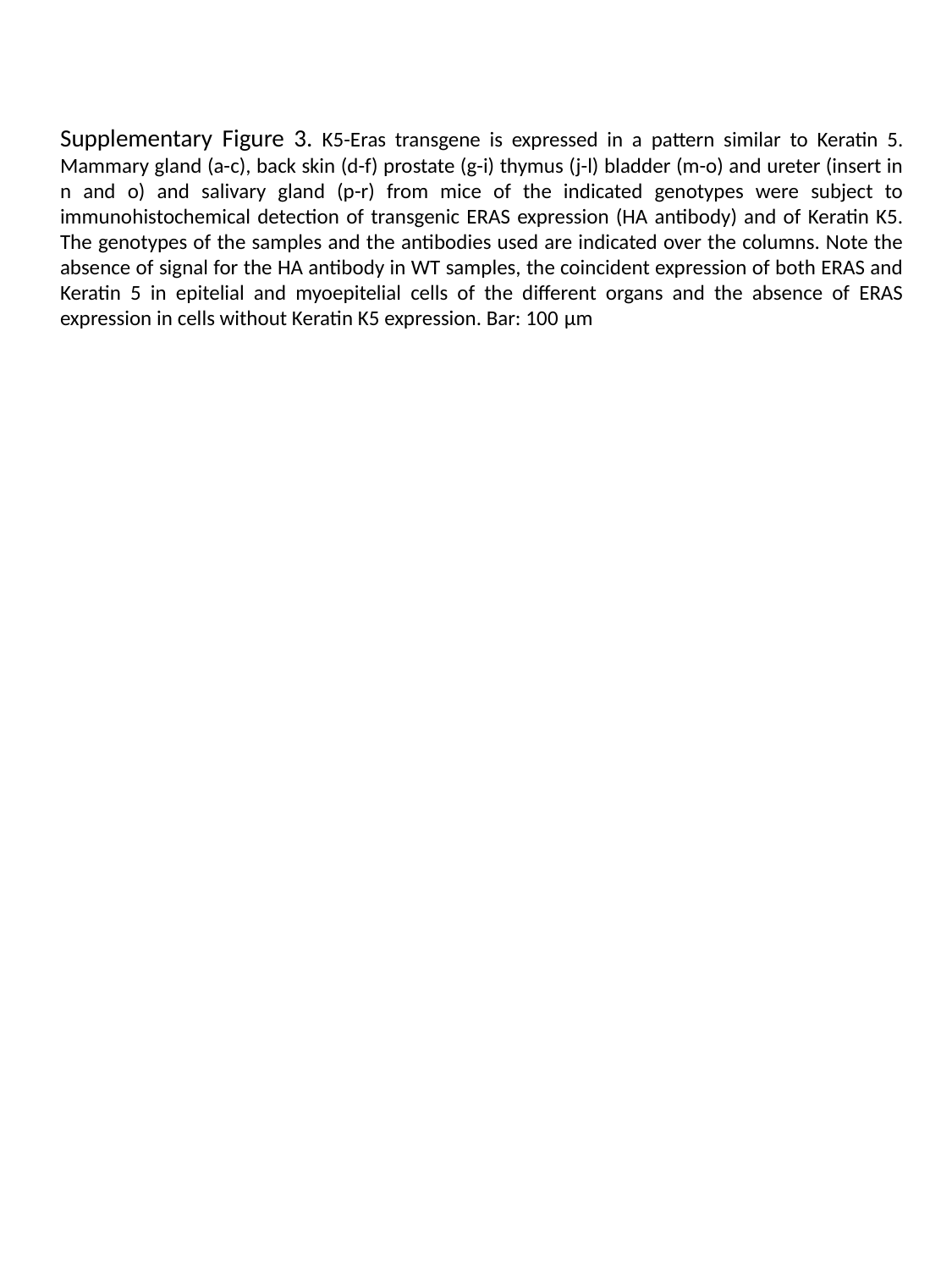

Supplementary Figure 3. K5-Eras transgene is expressed in a pattern similar to Keratin 5. Mammary gland (a-c), back skin (d-f) prostate (g-i) thymus (j-l) bladder (m-o) and ureter (insert in n and o) and salivary gland (p-r) from mice of the indicated genotypes were subject to immunohistochemical detection of transgenic ERAS expression (HA antibody) and of Keratin K5. The genotypes of the samples and the antibodies used are indicated over the columns. Note the absence of signal for the HA antibody in WT samples, the coincident expression of both ERAS and Keratin 5 in epitelial and myoepitelial cells of the different organs and the absence of ERAS expression in cells without Keratin K5 expression. Bar: 100 μm
