# Supplementary material for: ERAS, a Member of the Ras Superfamily, Acts as an Oncoprotein in the Mammary Gland
Source: Cancers (Basel). 2021 Nov 8;13(21):5588. doi: 10.3390/cancers13215588 (PMC8582886; doi:10.3390/cancers13215588)
Supplement: Supplementary file 1 [file cancers-13-05588-s001.zip › Supplementary Figure 4.pptx]

## Slide 1
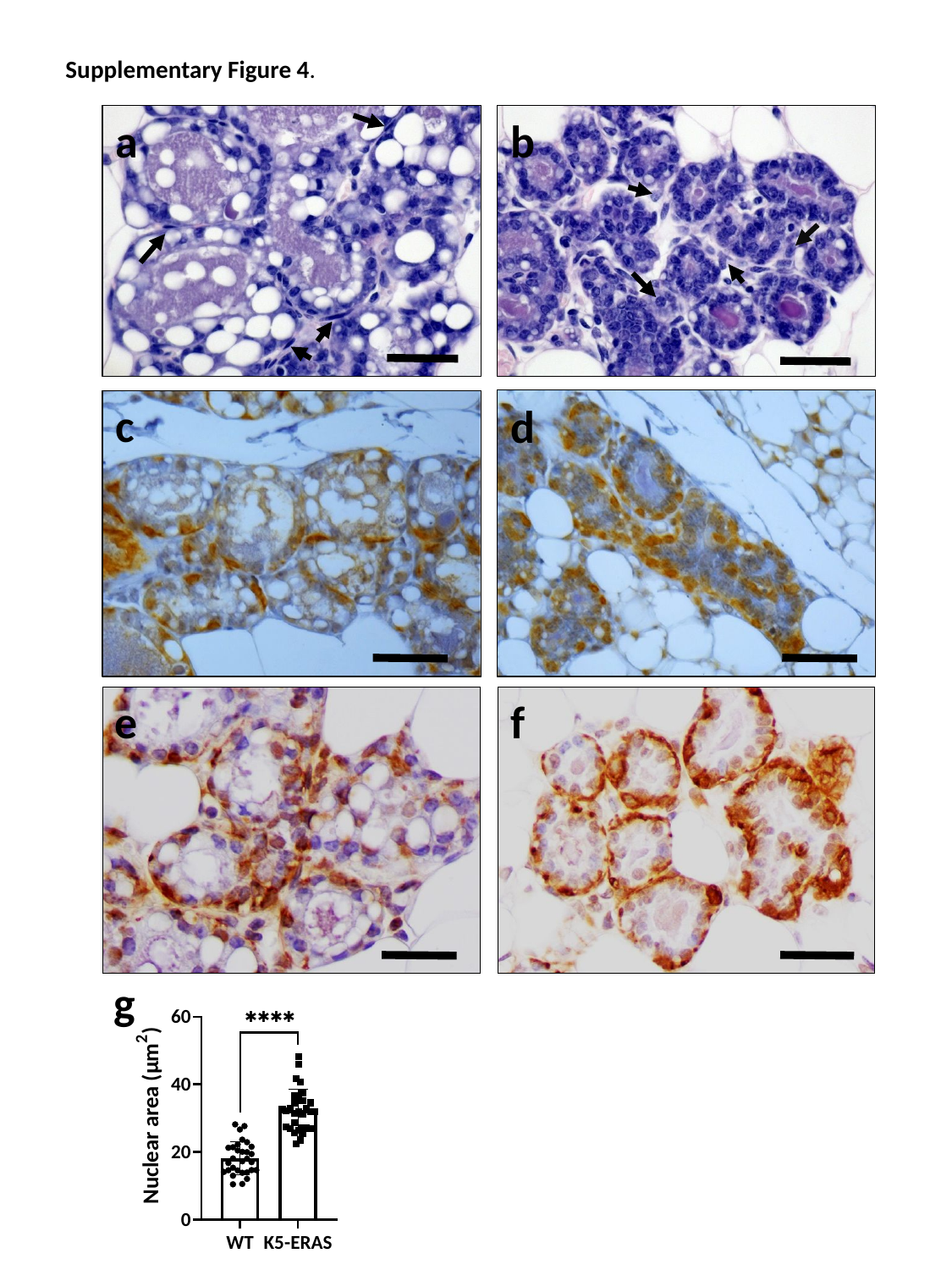

Supplementary Figure 4.
a
b
c
d
e
f
g

## Slide 2
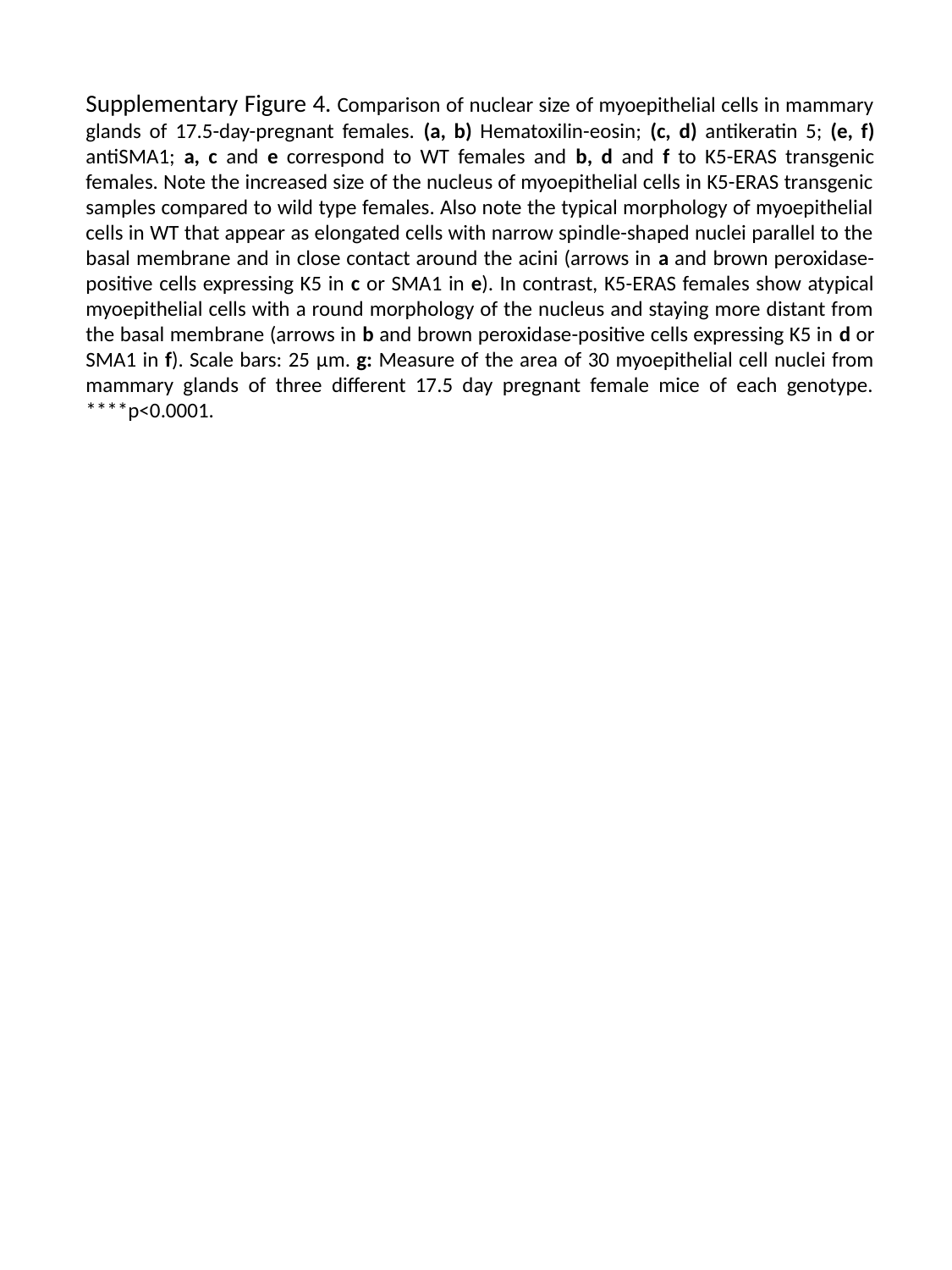

Supplementary Figure 4. Comparison of nuclear size of myoepithelial cells in mammary glands of 17.5-day-pregnant females. (a, b) Hematoxilin-eosin; (c, d) antikeratin 5; (e, f) antiSMA1; a, c and e correspond to WT females and b, d and f to K5-ERAS transgenic females. Note the increased size of the nucleus of myoepithelial cells in K5-ERAS transgenic samples compared to wild type females. Also note the typical morphology of myoepithelial cells in WT that appear as elongated cells with narrow spindle-shaped nuclei parallel to the basal membrane and in close contact around the acini (arrows in a and brown peroxidase-positive cells expressing K5 in c or SMA1 in e). In contrast, K5-ERAS females show atypical myoepithelial cells with a round morphology of the nucleus and staying more distant from the basal membrane (arrows in b and brown peroxidase-positive cells expressing K5 in d or SMA1 in f). Scale bars: 25 μm. g: Measure of the area of 30 myoepithelial cell nuclei from mammary glands of three different 17.5 day pregnant female mice of each genotype. ****p<0.0001.
