# Supplementary material for: ERAS, a Member of the Ras Superfamily, Acts as an Oncoprotein in the Mammary Gland
Source: Cancers (Basel). 2021 Nov 8;13(21):5588. doi: 10.3390/cancers13215588 (PMC8582886; doi:10.3390/cancers13215588)
Supplement: Supplementary file 1 [file cancers-13-05588-s001.zip › Supplementary Figure 5.pptx]

## Slide 1
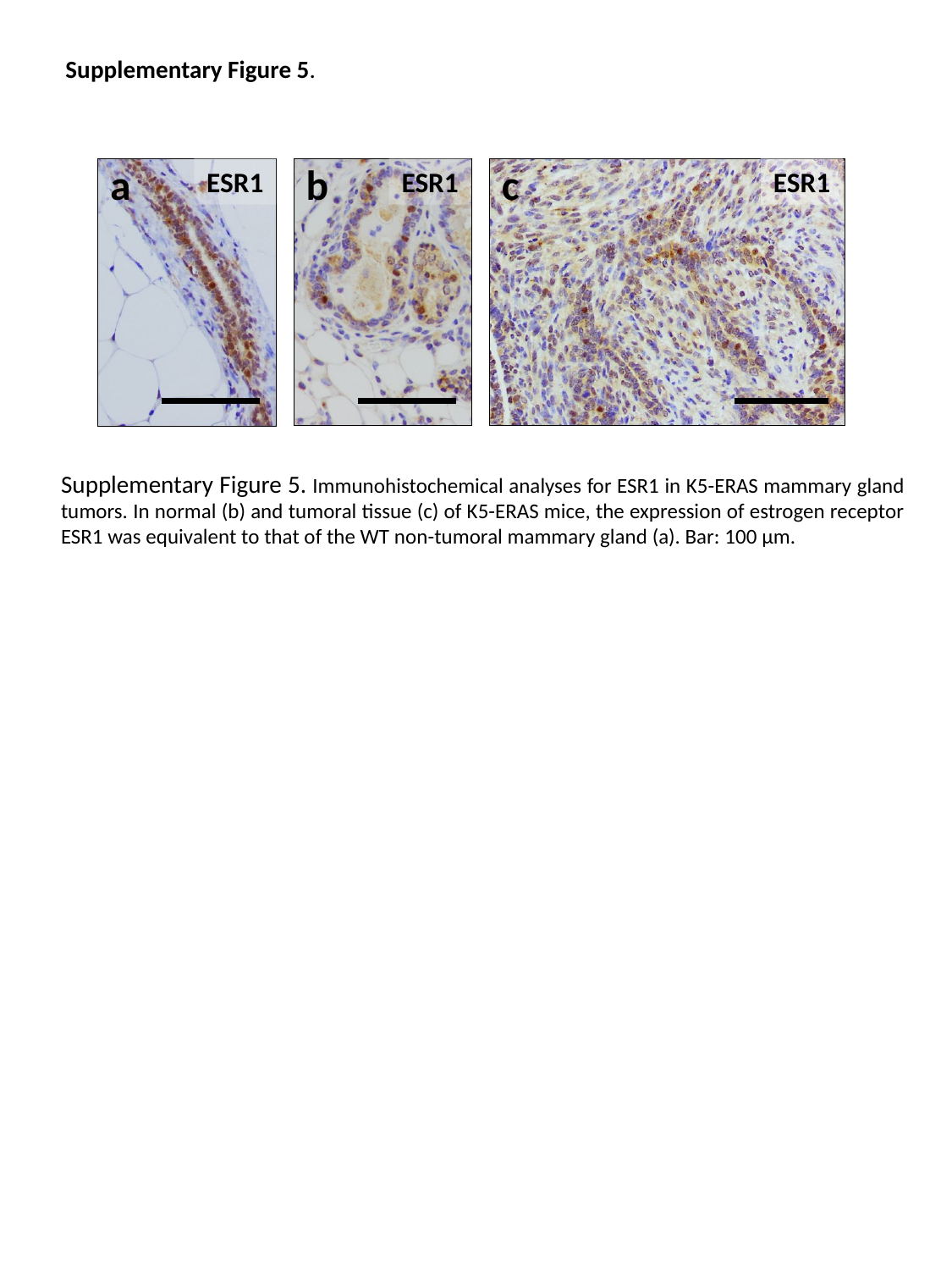

Supplementary Figure 5.
a
b
c
ESR1
ESR1
ESR1
Supplementary Figure 5. Immunohistochemical analyses for ESR1 in K5-ERAS mammary gland tumors. In normal (b) and tumoral tissue (c) of K5-ERAS mice, the expression of estrogen receptor ESR1 was equivalent to that of the WT non-tumoral mammary gland (a). Bar: 100 μm.
