# Supplementary material for: ERAS, a Member of the Ras Superfamily, Acts as an Oncoprotein in the Mammary Gland
Source: Cancers (Basel). 2021 Nov 8;13(21):5588. doi: 10.3390/cancers13215588 (PMC8582886; doi:10.3390/cancers13215588)
Supplement: Supplementary file 1 [file cancers-13-05588-s001.zip › Supplementary Figure 6.pptx]

## Slide 1
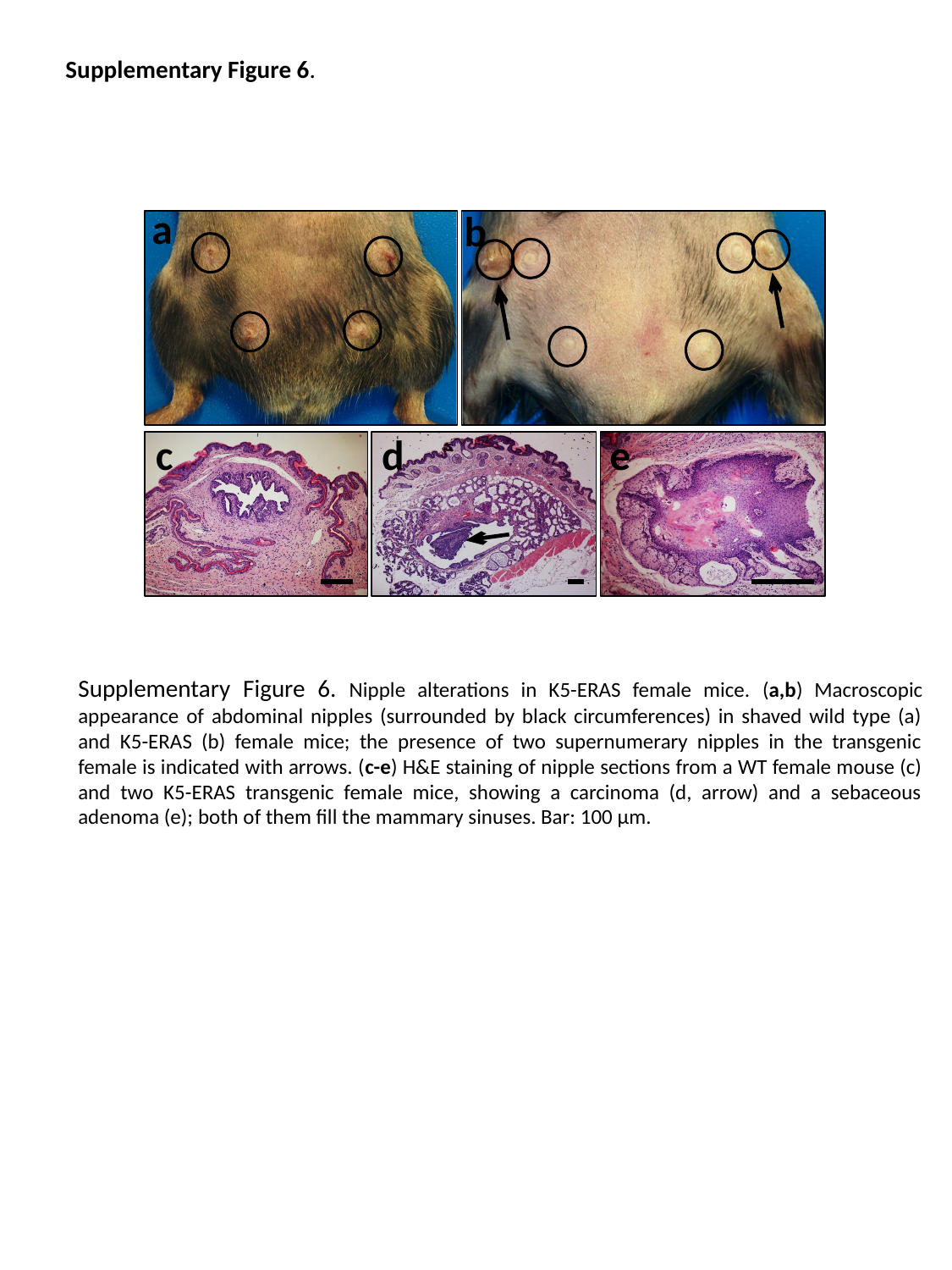

Supplementary Figure 6.
a
b
c
d
e
Supplementary Figure 6. Nipple alterations in K5-ERAS female mice. (a,b) Macroscopic appearance of abdominal nipples (surrounded by black circumferences) in shaved wild type (a) and K5-ERAS (b) female mice; the presence of two supernumerary nipples in the transgenic female is indicated with arrows. (c-e) H&E staining of nipple sections from a WT female mouse (c) and two K5-ERAS transgenic female mice, showing a carcinoma (d, arrow) and a sebaceous adenoma (e); both of them fill the mammary sinuses. Bar: 100 μm.
