# Supplementary material for: ERAS, a Member of the Ras Superfamily, Acts as an Oncoprotein in the Mammary Gland
Source: Cancers (Basel). 2021 Nov 8;13(21):5588. doi: 10.3390/cancers13215588 (PMC8582886; doi:10.3390/cancers13215588)
Supplement: Supplementary file 1 [file cancers-13-05588-s001.zip › Supplementary Figure 7.pptx]

## Slide 1
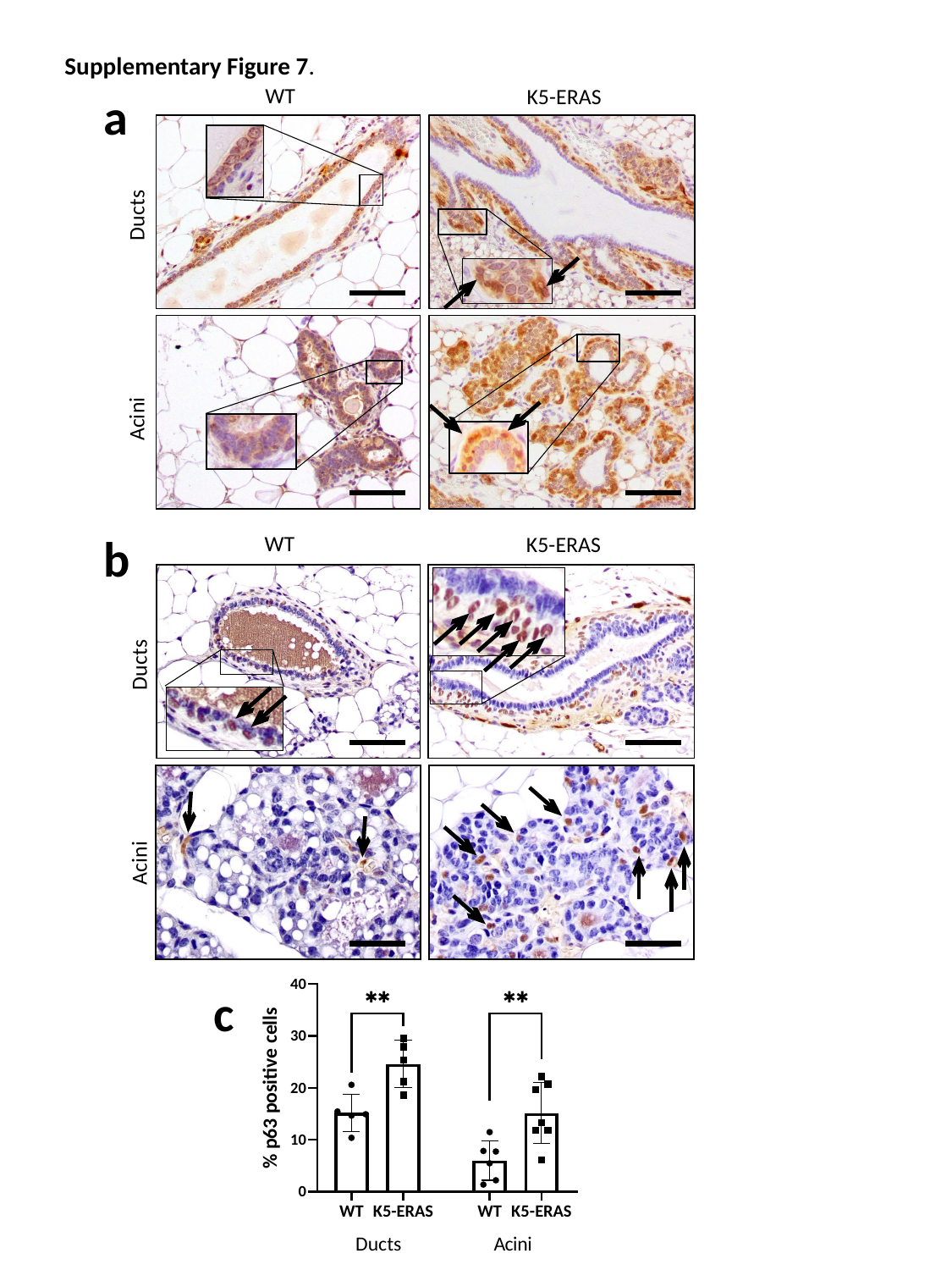

Supplementary Figure 7.
WT
K5-ERAS
Ducts
Acini
a
b
WT
K5-ERAS
Ducts
Acini
c

## Slide 2
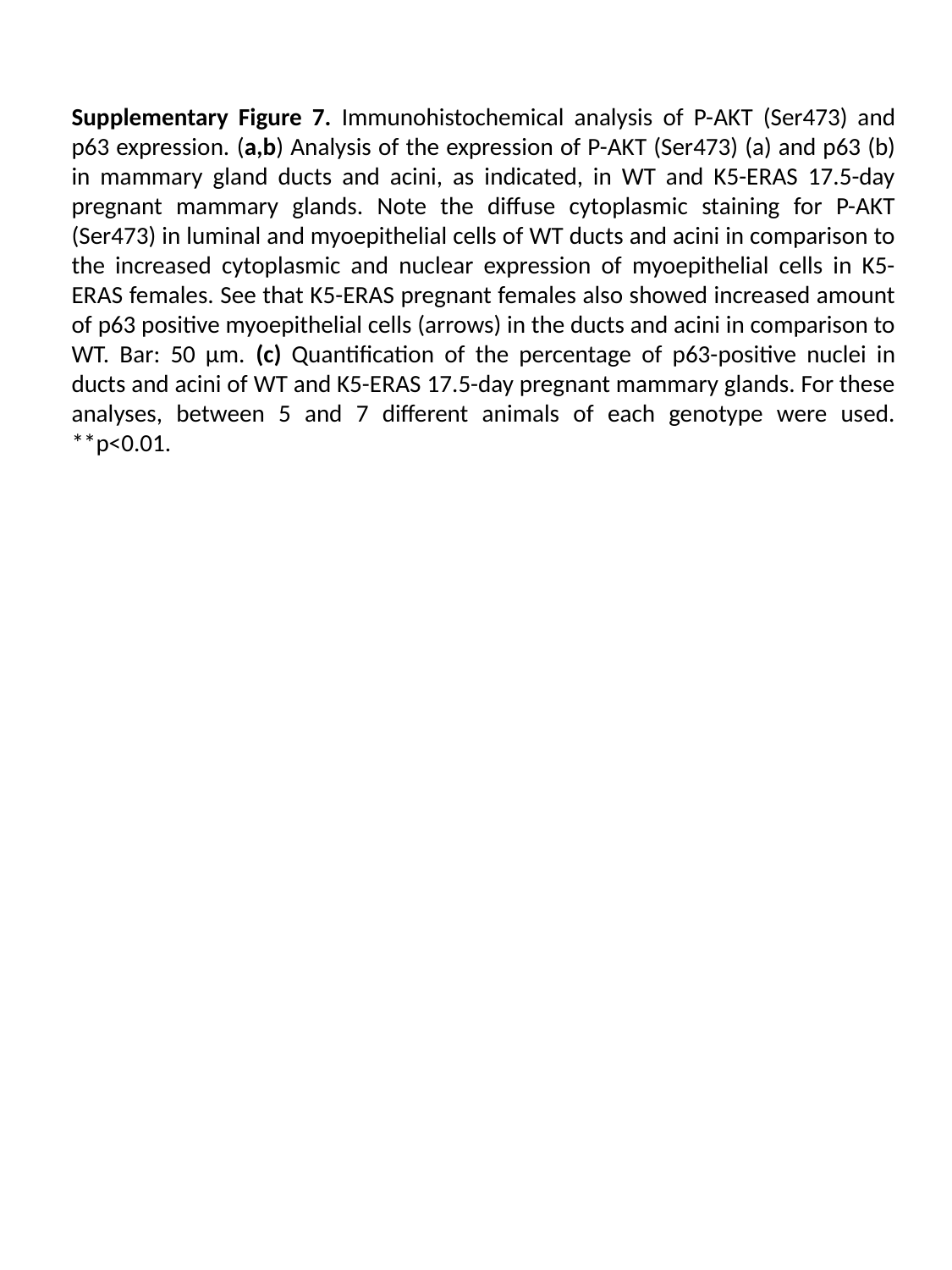

Supplementary Figure 7. Immunohistochemical analysis of P-AKT (Ser473) and p63 expression. (a,b) Analysis of the expression of P-AKT (Ser473) (a) and p63 (b) in mammary gland ducts and acini, as indicated, in WT and K5-ERAS 17.5-day pregnant mammary glands. Note the diffuse cytoplasmic staining for P-AKT (Ser473) in luminal and myoepithelial cells of WT ducts and acini in comparison to the increased cytoplasmic and nuclear expression of myoepithelial cells in K5-ERAS females. See that K5-ERAS pregnant females also showed increased amount of p63 positive myoepithelial cells (arrows) in the ducts and acini in comparison to WT. Bar: 50 μm. (c) Quantification of the percentage of p63-positive nuclei in ducts and acini of WT and K5-ERAS 17.5-day pregnant mammary glands. For these analyses, between 5 and 7 different animals of each genotype were used. **p<0.01.
