# Supplementary material for: ERAS, a Member of the Ras Superfamily, Acts as an Oncoprotein in the Mammary Gland
Source: Cancers (Basel). 2021 Nov 8;13(21):5588. doi: 10.3390/cancers13215588 (PMC8582886; doi:10.3390/cancers13215588)
Supplement: Supplementary file 1 [file cancers-13-05588-s001.zip › Supplementary Figure 8.pptx]

## Slide 1
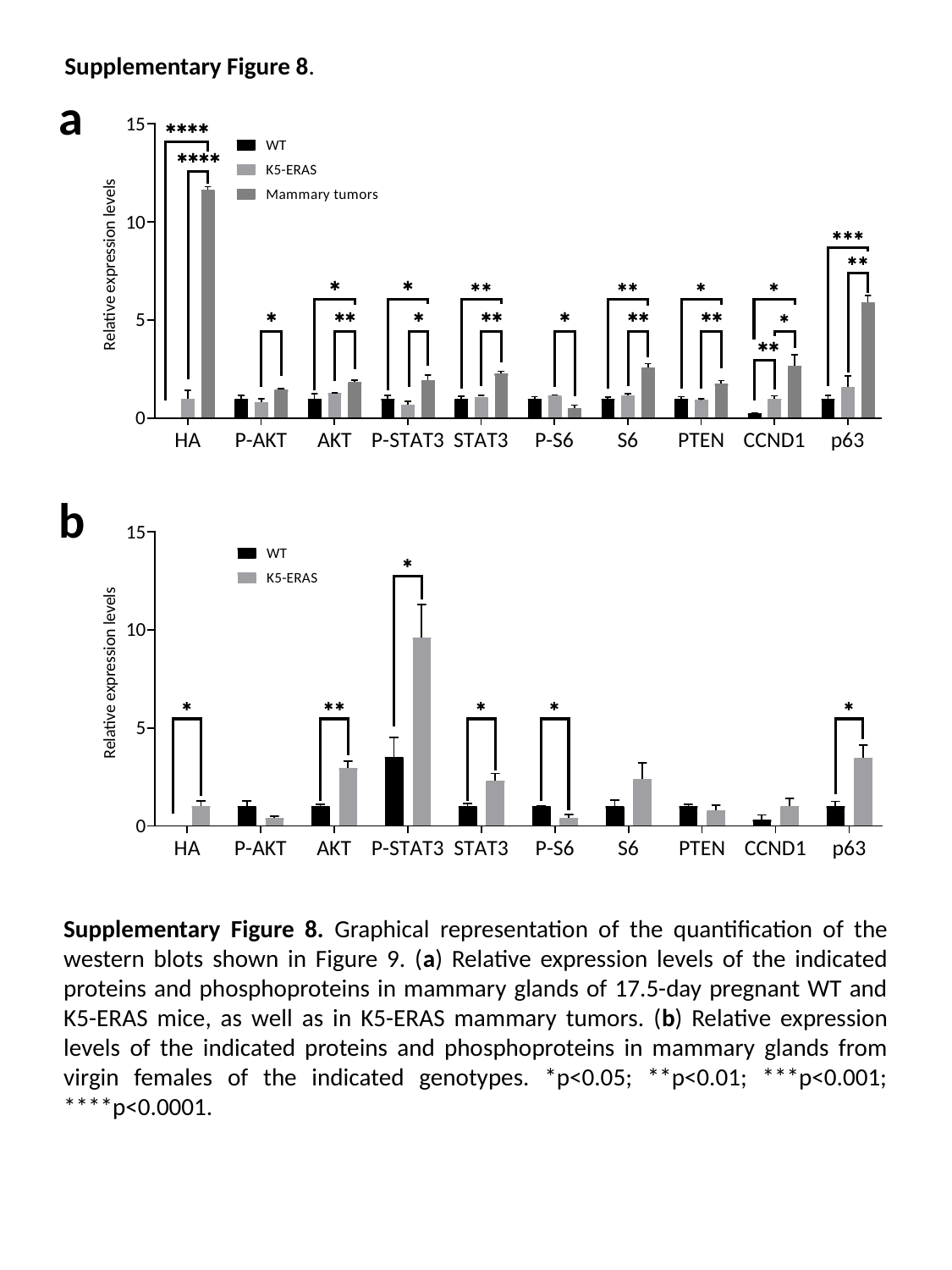

Supplementary Figure 8.
a
b
Supplementary Figure 8. Graphical representation of the quantification of the western blots shown in Figure 9. (a) Relative expression levels of the indicated proteins and phosphoproteins in mammary glands of 17.5-day pregnant WT and K5-ERAS mice, as well as in K5-ERAS mammary tumors. (b) Relative expression levels of the indicated proteins and phosphoproteins in mammary glands from virgin females of the indicated genotypes. *p<0.05; **p<0.01; ***p<0.001; ****p<0.0001.
